# The Structural Features of Novel Bacterial Topoisomerase Inhibitors That Define Their Activity on Topoisomerase IV

**DOI:** 10.1021/acs.jmedchem.2c00039

**Published:** 2022-05-03

**Authors:** Maja Kokot, Marko Anderluh, Martina Hrast, Nikola Minovski

**Affiliations:** † Theory Department, Laboratory for Cheminformatics, National Institute of Chemistry, Hajdrihova 19, SI-1001 Ljubljana, Slovenia; ‡ Department of Pharmaceutical Chemistry, Faculty of Pharmacy, University of Ljubljana, Aškerčeva cesta 7, SI-1000 Ljubljana, Slovenia

## Abstract

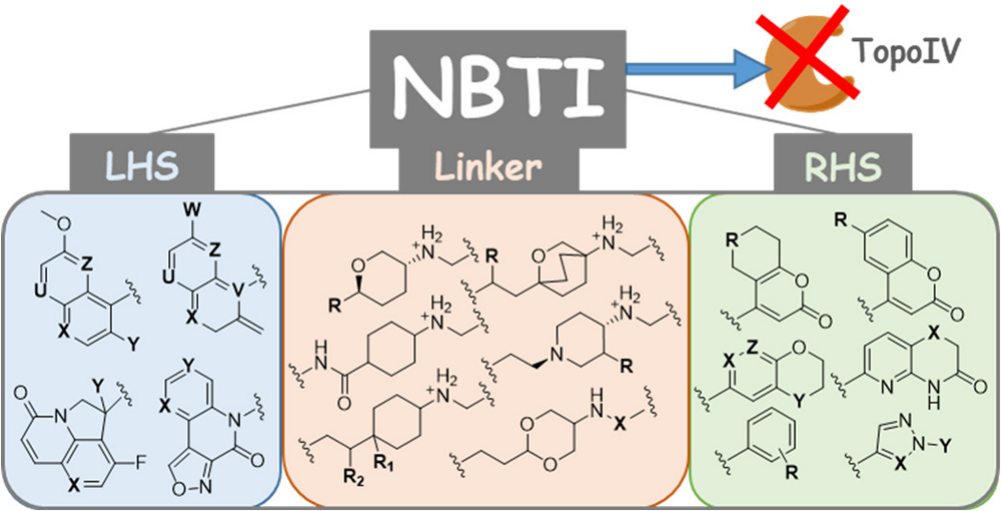

The continued emergence of bacterial resistance has created an urgent need for new and effective antibacterial agents. Bacterial type II topoisomerases, such as DNA gyrase and topoisomerase IV (topoIV), are well-validated targets for antibacterial chemotherapy. The novel bacterial topoisomerase inhibitors (NBTIs) represent one of the new promising classes of antibacterial agents. They can inhibit both of these bacterial targets; however, their potencies differ on the targets among species, making topoIV probably a primary target of NBTIs in Gram-negative bacteria. Therefore, it is important to gain an insight into the NBTIs key structural features that govern the topoIV inhibition. However, in Gram-positive bacteria, topoIV is also a significant target for achieving dual-targeting, which in turn contributes to avoiding bacterial resistance caused by single-target mutations. In this perspective, we address the structure–activity relationship guidelines for NBTIs that target the topoIV enzyme in Gram-positive and Gram-negative bacteria.

## Introduction

In recent years, multidrug-resistant infections caused by Gram-positive organisms such as methicillin-resistant *Staphylococcus aureus*(1) have become a serious threat, with more than 10,600 deaths per year according to a 2019 report by the Centers for Disease Control and Prevention (CDC).^2^ Gram-negative bacteria such as *Klebsiella pneumoniae*, *Acinetobacter baumannii*, *Pseudomonas aeruginosa*, and *Enterobacter* spp. also prompt a special attention due to their permeability barrier and potentiated efflux pumps that expel compounds.^3^ These bacteria, including *Enterococcus faecium* and *S. aureus*, belong to the class of “ESKAPE” pathogens, nowadays portrayed as highly virulent and antibiotic resistant.^4^ They represent a particular burden in hospitals for patients with compromised immune systems.^5^ In addition, classifications from the World Health Organization (WHO) and CDC categorize pathogens similar to ESKAPE as a serious health concern, for which new antibacterial agents should be discovered. It is estimated that 4.95 million deaths were associated with antimicrobial resistance in 2019, and 1.27 million of the deaths were attributed to it.^6^ Considering the current situation, it is estimated that by 2050, the number of human deaths caused by bacterial infections will exceed 10 million per year, and the majority of these will probably be the consequence of antimicrobial resistance.^7^

Among the various antibacterial targets, bacterial type II topoisomerases, such as DNA gyrase and its paralogous counterpart topoisomerase IV (topoIV), have been distinguished as well-established and clinically important targets for antibacterial agents. These heterotetrameric enzymes consist of two GyrA and two GyrB subunits in DNA gyrase (i.e., A_2_B_2_) and two ParC and two ParE subunits in topoIV (i.e., C_2_E_2_).^8^ Despite their high levels of structural similarity, they are involved in different intracellular functions in bacteria.^9^ DNA gyrase introduces negative supercoils into the DNA molecule and removes positive supercoils that accumulate ahead of replication forks and transcription complexes, while topoIV is responsible for removal of DNA knots and decatenation of tangles generated during recombination and replication.^8−12^ Nearly two decades ago, there were mainly two widely known classes of inhibitors targeting bacterial topoisomerases type II: fluoroquinolones that target the catalytic domains (GyrA and ParC in DNA gyrase and topoIV, respectively) and aminocoumarins that target the ATPase domains (GyrB and ParE in DNA gyrase and topoIV, respectively).^10^ However, due to the solubility and toxicity issues of aminocoumarins,^10^ fluoroquinolones are among the best topoisomerase-targeting inhibitors that have been widely used in antibacterial chemotherapy for more than 50 years.^13^ Notwithstanding their high therapeutic success, they suffer from lack of activity, mainly due to target-based mutations and consequent bacterial resistance.^14^ Therefore, there is an urgent need to develop new topoisomerase-targeting antibacterial agents that lack cross-resistance to the existing 6-fluoroqinolones.^14−16^

Consequently, two new classes of non-ATPase, nonquinolone class of bacterial topoisomerase inhibitors targeting DNA gyrase/topoIV enzymes have recently been discovered, which include the so-called “novel bacterial topoisomerase inhibitors” (*alias* NBTIs)^17−23^ and the quinolinepyrimidinetriones or spiropyrimidinetriones.^24^ The latter ones have been evolved from the lead compound QPT-1 (PNU-286607), originally discovered by Pharmacia.^25^ A representative of this class is zoliflodacin (ETX0914), which is currently in Phase 3 clinical trials for the treatment of infections caused by *Neisseria gonorrheae*. This antibacterial agent has a binding mode that is completely distinct from that of both the quinolones and the NBTIs. However, relative to the fluoroquinolones that target both GyrA/GyrB subunits in DNA gyrase and ParC/ParE subunits in topoIV, the primary targets of zoliflodacin are solely the DNA gyrase GyrB and topoIV ParE subunit. Consequently, there is no target-based cross-resistance with the quinolone class of antibacterial agents.^26^

NBTIs were first discovered by GlaxoSmithKline and Aventis Pharma AG.^19,20^ Viquidacin (NXL-101), an NBTI discovered by Aventis and developed by Novexel, underwent phase I clinical trials; however, it was discontinued due to its hERG-related cardiotoxic issues manifested as prolonged QT signals in the heart.^27^ The most advanced NBTI is gepotidacin (GSK2140944), which is currently in the third phase of clinical trials for the treatment of uncomplicated urogenital gonorrhea caused by *N. gonorrheae*(28,29) as well as uncomplicated urinary tract infections (e.g., acute cystitis) most frequently caused by *Escherichia coli*.^30,31^ Gepotidacin exhibits a well-balanced, dual-targeting inhibition of DNA gyrase and topoIV in *N. gonorrheae*. During the treatment with gepotidacin, two point mutations were observed, that is, a pre-existing ParC D86N and an additional GyrA A92T mutation. These mutations alone did not significantly weaken the antibacterial activity, while their concomitant presence results in more than 16-fold reduction in the antibacterial activity.^28,32^ It is also worth mentioning that gepotidacin completed phase II of clinical trials for the treatment of Gram-positive acute bacterial skin and skin structure infections.^33^ Similarly to *N. gonorrheae*, two point mutations (e.g., ParC V67A and GyrA D83N) were observed in *S. aureus* topoisomerases, which have not been shown to pre-exist in *S. aureus* clinical isolates.^33^ This clearly implicates that the primary target of gepotidacin in Gram-positive bacteria is DNA gyrase, while in Gram-negative organisms, it is topoIV, as for the majority of NBTIs.^33,34^ Notwithstanding the positive microbiological results of gepotidacin in treatments of infections caused by *S. aureus*,^33^ unfortunately there are no additional studies performed to date.

**Figure 1:**
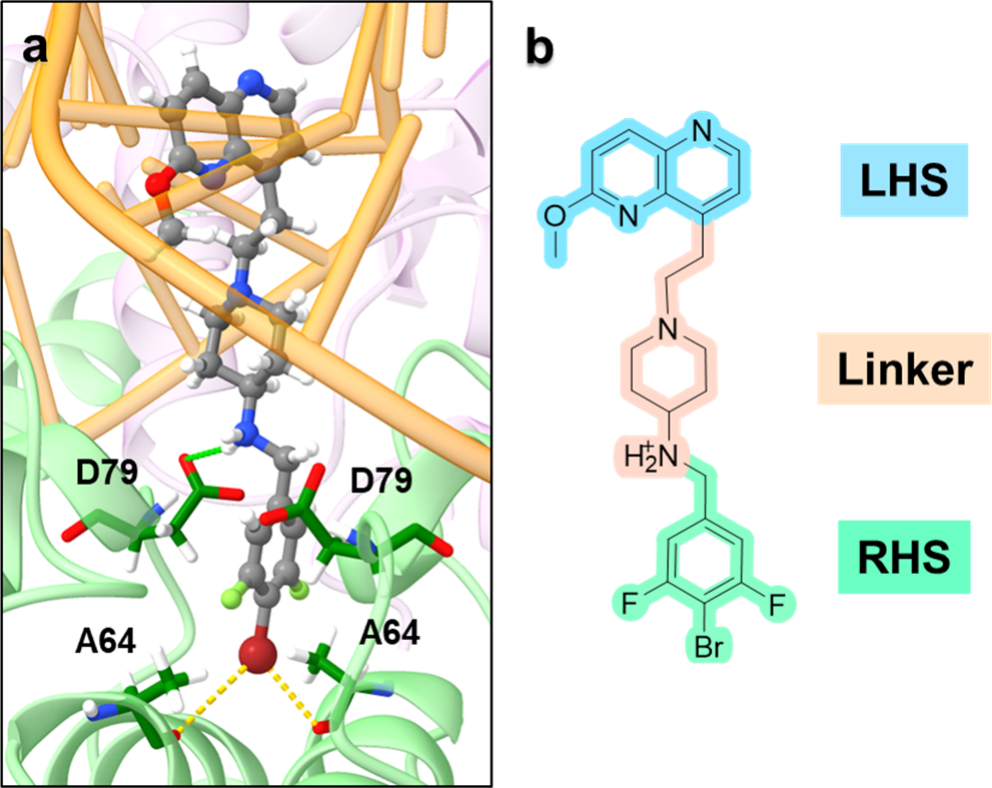
(a) An *E. coli* topoIV protein homology model showing a predicted binding pose of an NBTI (compound **8** from Kokot et al.^41^). The topoIV enzyme and DNA are represented as cartoons (ParC subunit in green, ParE subunit in purple, DNA in orange). The NBTI is in a ball and stick representation in gray. The halogen bonds are shown as yellow dots and ionic interactions as green dots. (b) Two-dimensional structure of a representative NBTI comprised of the LHS, linker, and RHS moieties.

NBTIs consist of three structural moieties: a bicyclic or tricyclic heteroaromatic “left-hand side” (LHS; Figure 1b, blue) that intercalates between the central DNA base pairs; a bicyclic or monocyclic heteroaromatic “right-hand side” (RHS; Figure 1b, green) that binds into a deep, noncatalytic hydrophobic binding pocket assembled by the two GyrA and ParC subunits of DNA gyrase and topoIV (Figure 1a), respectively; and a cyclic/bicyclic linker moiety (Figure 1b, orange) that connects both sides to provide the correct spatial geometry of the ligand.^17,21,22^ Compared to fluoroquinolones, NBTIs bind to DNA gyrase and topoIV at an alternative binding site^34^ that is formed at the interface of the two DNA gyrase GyrA and topoIV ParC subunits before the cleavage of the DNA.^22,35^

The primary target of NBTIs in Gram-positive bacterial pathogens (e.g., *S. aureus*) is DNA gyrase. However, it was demonstrated that whether the potency on *S. aureus* topoIV is low, the point mutations in DNA gyrase of the same organism (e.g., D83N) can significantly contribute in reducing the NBTIs antibacterial activities, as well.^36,37^ On the contrary, in Gram-negative bacteria, it seems that topoIV might be a primary target, as exemplified by the *in vitro* enzyme inhibitory data for *E. coli*.^37,38^ These findings are in agreement with the observations of Nayar et al., according to which point mutations in the individual enzymes, such as D82G in *E. coli* DNA gyrase or D79G in *E. coli* topoIV, do not result in decreased antibacterial activity, while the concomitance of these point mutations in both enzymes can cause a 128-fold decrease in the antibacterial activity.^39^ Moreover, a recent study revealed that some compounds show similar inhibitory potencies against the D83N mutant of *S. aureus* DNA gyrase as against topoIV from the same organism. It was also confirmed that NBTIs with superior dual-targeting inhibition of *S. aureus* topoIV and *S. aureus* DNA gyrase D83N mutant show low frequencies of resistance.^40^

In this perspective, we address the structure–activity relationship (SAR) guidelines for NBTIs that target the topoIV enzyme in Gram-positive and Gram-negative bacteria. The structural differences of each of the structural moieties (i.e., LHS, linker, RHS) comprising a comprehensive set of NBTI representatives included in this perspective are thoroughly described, and their impact on the inhibition of topoIV activity is also discussed. Due to the unavailability of any experimental data of topoIV in complex with DNA and NBTI (e.g., no X-ray or cryo-EM structure), the inhibitory potencies of NBTI representatives (IC_50_ values) discussed here were compared with their structural features. We are aware that the different assay formats for determining topoIV inhibition by the NBTIs reviewed in this perspective are not directly comparable to each other. For instance, the topoIV decatenation assay was performed in refs (19, 36,40, 42−47), the DNA topoIV relaxation assay was used in refs (19, 37, 38, 41, 48, and 49), while the ATPase activity of *E. coli* ParE subunit was determined in refs (39 and 50−53). The comparison of two different assays (e.g., DNA Cleavage and topoIV DNA Decatenation) for the same compounds^19,54^ showed that the relative potency of a given compound can be different depending on the assay used. Therefore, we compared here the individual NBTI’s structural features and the impact that they have on the topoIV enzyme inhibition performed by the same research groups and by using the same assay protocols.

## The Left-Hand Side Moiety

The LHS moiety of NBTIs intercalates between the central DNA base pairs in a similar fashion as for DNA gyrase. The most commonly used LHS fragments are variously substituted bicyclic heteroaromatic systems, such as quinolines, quinoxalines, and naphthyridines.^37,42−44,47,49−53^

**Figure 2:**
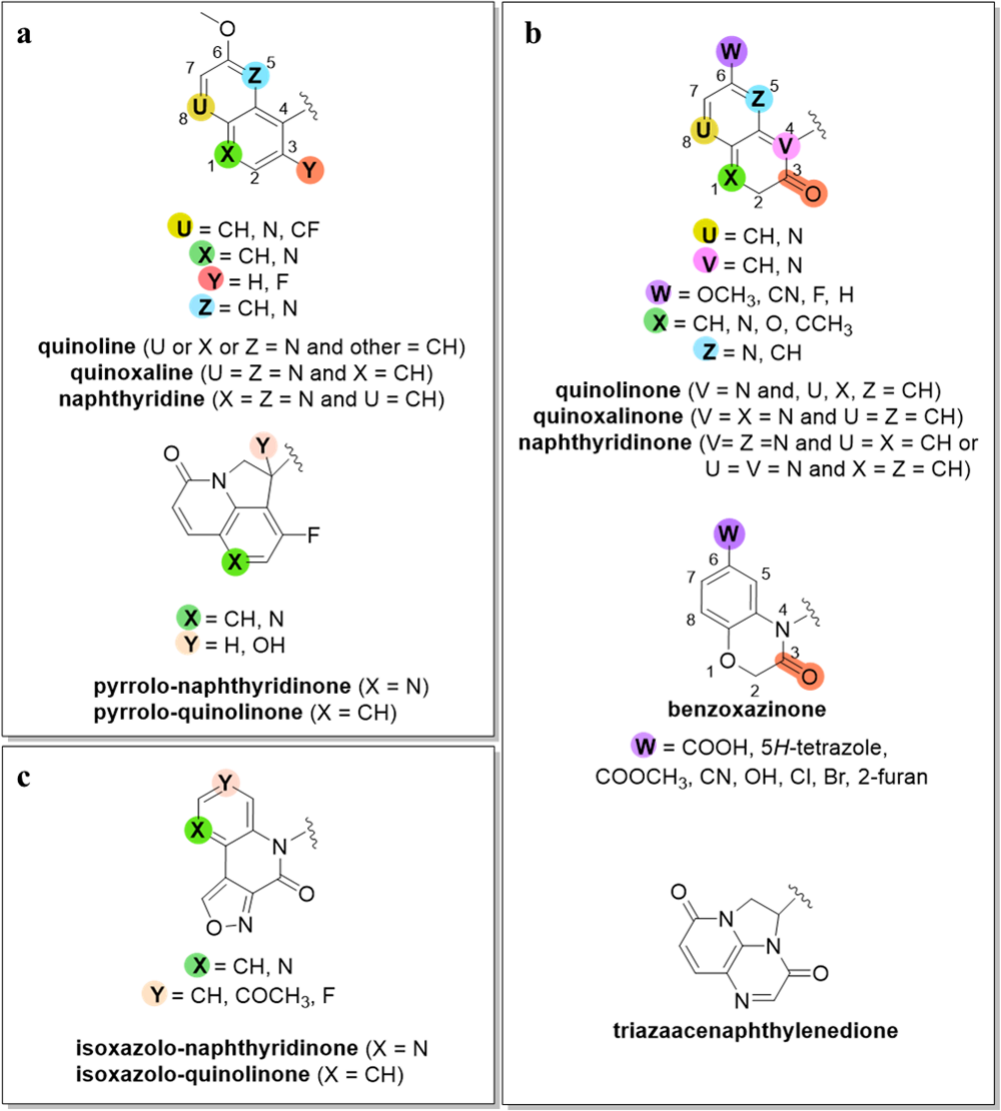
SAR guidelines for LHS moiety derivatives: (a) quinoline, quinoxaline, naphthyridine, and pyrrolo derivative; (b) quinolinone, quinoxalinone, naphthyridinone, benzoxazinone, and triazaacenaphthylenedione; and (c) isoxazolo derivative.

In general, LHS constructs comprising small substituents at position six (Figure 2b, purple) have been demonstrated to be preferred.^53^ Compared to the unsubstituted six position of LHS constructs, the 6-methoxy variant has stronger topoIV inhibitory activity. Cyano and fluoro substituents at position six are suitable alternatives to a methoxy group.^50,53^ Larger groups, such as carboxylic acid and its bioisosteric replacement tetrazole or ester, have negative effects on the NBTI inhibition of topoIV activity in *E. coli* (Table S1, **1**), while small substitutions (e.g., −OH, −CN, −Cl, −Br) result in increased potencies (Table S1, **2**).^53^

Fluorination is a commonly explored strategy in medicinal chemistry that affects not only physicochemical properties but also the absorption, distribution, metabolism, excretion, and toxicity (ADMET) profile of compounds.^55^ There are numerous NBTIs that include 3-fluoro quinoline, quinoxaline, or naphthyridine LHS moieties (Figure 2a, red) that exhibit improved topoIV inhibition over their unsubstituted analogs (Table S1, **3** and **4**).^36,42,48,49^

To restrain the conformational rotation of LHS in the ethyl bridge, a carbonyl group was introduced at position three (Figure 2b, red). Depending on the naphthyridine LHS constructs used, the replacement of fluorine with a carbonyl group results in differences in the topoIV inhibition. Put differently, the replacement of 3-fluoro-1,5-naphthyridine LHS moiety (Table S1, **5** and **8**) with 2-oxo-1,8-naphthyridine LHS (Table S1, **6** and **9**) does not have an effect on *E. coli* topoIV inhibition (Table S1, **5**/**6** and **8**/**9**).^48−50^ Moreover, the replacement of 3-fluoro-1,5-naphthyridine LHS moiety (Table S1, **5** and **10**) with 2-oxoquinoxaline LHS (Table S1, **7** and **11**) resulted in the loss of *S. aureus* topoIV inhibition (Table S1, **10**/**11**) and reduction of inhibition of *E. coli* topoIV (Table 1, **5**/**7**).^43,50^ It should also be stressed that the replacement of fluorine by carbonyl always includes the change of C- to N-linked LHS variants.^43,48−50^

It appears that for the numbers and positions of the nitrogens in bicyclic aromatic LHS variants, there is no strict correlation between the structural changes and the topoIV inhibition. Hence, the NBTI inhibitory potency depends on the various LHS substitutions. Put differently, in Gram-positive bacteria (e.g., *S. aureus*), variously substituted quinoline LHS moieties can diversely impact the potencies for enzyme inhibition, compared to Gram-negative bacteria (e.g., *E. coli*), where different nitrogen positions in the quinoline LHS fragment can result in the same potencies for topoIV inhibition (Table S1, **12**–**14**).^37^ For targeting *E. coli*, the three most studied bicyclic LHS variants have been quinoline, quinoxaline, and naphthyridine (Figure 2a) with fluorine substitution at position three, that is, a carbonyl group at the same position as for quinolinone, quinoxalinone, and naphthyridinone (Figure 2b). However, in the case of *S. aureus*, only 3-fluorinated LHS constructs have been studied, which have shown similar inhibitory potencies (Table S1, **8**, **15**, and **16**).^48^ It appears that in some cases, the naphthyridine LHS moiety can slightly improve the inhibitory potency compared to NBTI analogs with quinoline or quinoxaline LHS moieties (Table S1, **17**–**19**).^37^ Nevertheless, NBTIs with quinoxalinone and quinolinone LHS fragments show stronger inhibitory potencies compared to the naphthyridinone containing analogs (Table S1, **7**, **20**, and **21**).^50^ It was reported, however, that in all cases, bicyclic LHS moieties that contain more than two nitrogens show significantly decreased potencies for enzyme inhibition. Moreover, the NBTIs comprising 6-oxo-naphthyridine LHS construct with 2-hydroxyethyl substitution at position 5 showed similar topoIV inhibition for both bacterial strains (Table S1, **22**).^56^

It is also important to note that the replacement of the benzoxazinone LHS scaffold with a dihydroquinolinone moiety (Table S1, **23** and **24**) results in an enormous loss of topoIV inhibition. In a similar fashion, replacement of the lactam with a urea group (Table S1, **21** and **25**) can also lead to decreased potency for *E. coli* enzyme inhibition.^50^

It has been shown that introduction of an additional nitrogen atom into the 2-aza quinolinone LHS is also tolerated. LHS variants with aza substitution at positions 1 or 5 can lead to increased inhibitory potency, while introduction of nitrogen at positions 4 or 7 results in decreased potency.^50^

Various tricyclic LHS compounds have also been studied, such as triazaacenaphthylenedione, pyrrolo-naphthyridinone, pyrrolo-quinolinone, and isoxazolo-quinolinone (Figure 2a–c).^36,37,43,44,47^ The inhibitory potencies of such NBTI derivatives against topoIV mainly depend on the type of tricyclic LHS moiety. For instance, a tricyclic quinolone LHS can lead to loss of *S. aureus* topoIV inhibition in some cases, as exemplified by the compounds **11** and **26** (Table S1).^43^ However, in case of *E. coli* topoIV inhibition, compounds with tricyclic LHSs express potencies in low micromolar concentrations (Table S1, **27**).^47^

It should be pointed out that although the NBTIs LHS fragment intercalates between the central DNA base pairs without making any direct contact with the enzymes (Figure 1a), it also importantly effects NBTI’s topoIV inhibition (Table S1, **13**–**18**), via altering the overall ligand’s physicochemical profile as well as via different intercalation between DNA base pairs. Namely, the way LHS intercalates between DNA base pairs might affect the spatial orientation of the rest of NBTI molecule. This cannot be confirmed without a doubt because no structural data on topoIV in complex with an NBTI are available. However, superposition of the cocrystallized NBTI ligands from related DNA gyrase/DNA/NBTI complexes (Figure S1) clearly shows that the position of the intercalated LHS affects the spatial orientation of the linker and consequently guides how the RHS fragment fits into the enzyme binding pocket. Since LHS intercalates in a similar fashion between central DNA base pairs in DNA gyrase and topoIV, we expect a similar influence on the RHSs binding to topoIV. *E. coli* topoIV is not as sensitive to variations in LHS as *S. aureus* (Table S1, e.g., **13**/**14** and **17**/**18**); however, this is not always a case, as evidenced by the comparison of compounds **15**/**16** (Table S1). Again, the lack of structural data for topoIV in complex with an NBTI prevents a structural explanation of such experimental results. Therefore, there is an urgent need for experimental data for topoIV in complex with DNA and NBTI.

## The Linker

**Figure 3:**
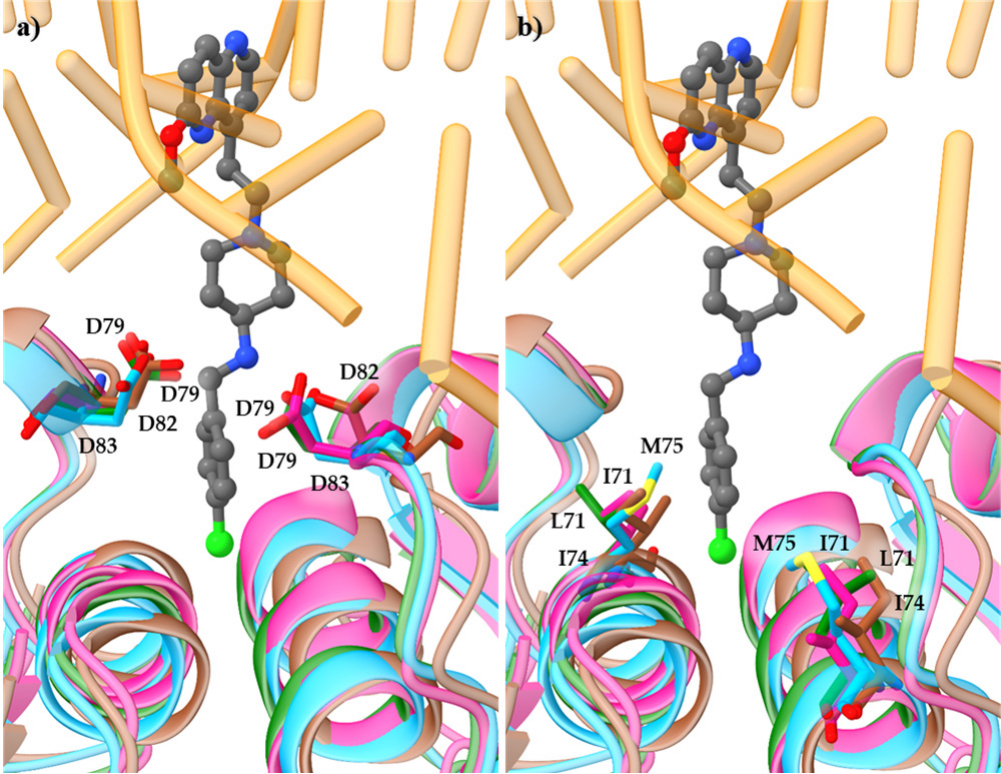
Structural alignment of DNA gyrase and topoIV enzymes originating from *S. aureus* and *E. coli*, respectively, together with the cocrystallized NBTI AMK-12 ligand from *S. aureus* DNA gyrase complex (PDB ID: 6Z1A;^22^Table S1, **46**). *S. aureus* DNA gyrase (cyan), *E. coli* DNA gyrase (brown, PDB ID: 6RKS),^58^*S. aureus* topoIV homology model (pink), and *E. coli* topoIV homology model (geren). The enzymes are represented as cartoon, NBTI ligand **46** as sticks, DNA in orange, (a) GyrA/ParC Met, Ile, and Leu residues as sticks colored by element, and (b) GyrA/ParC aspartate residues as sticks colored by element.

**Figure 4:**
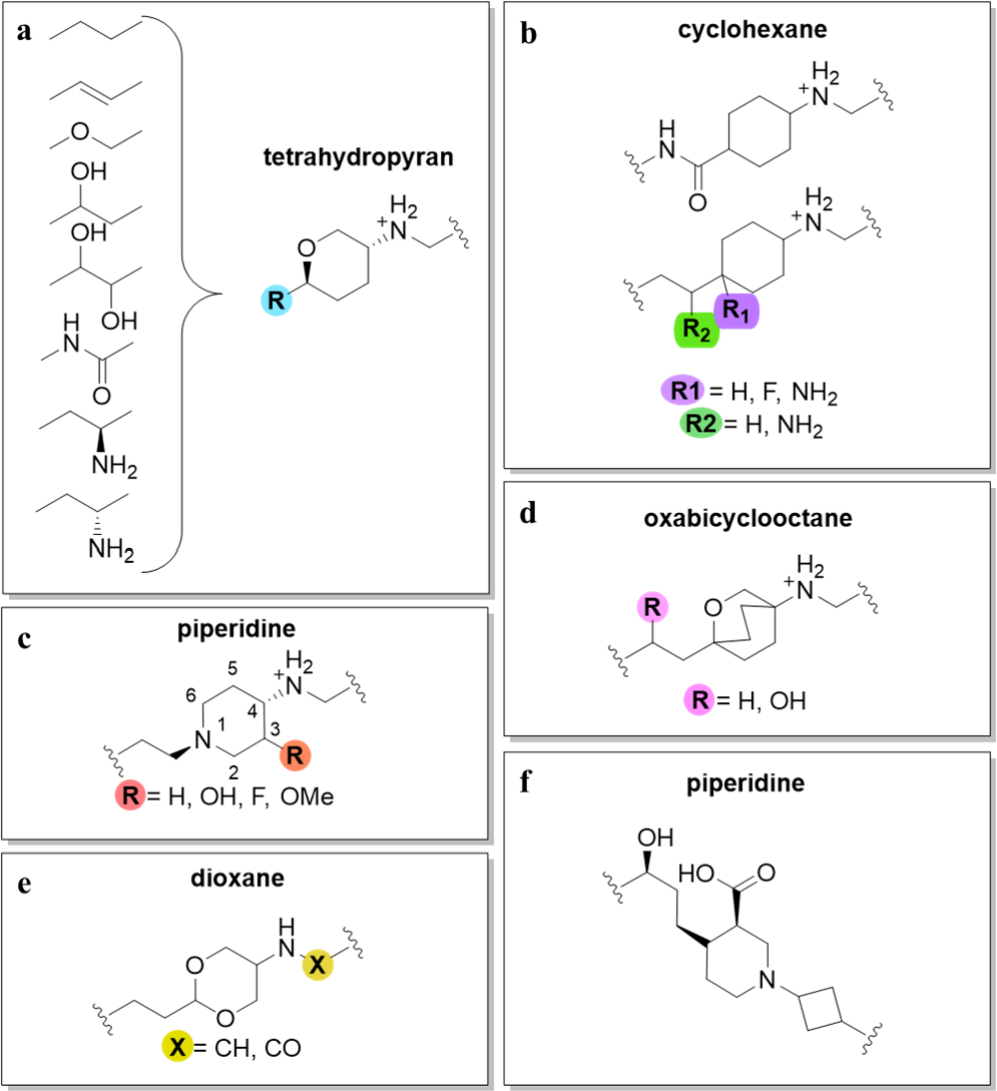
SAR guidelines for the linker moiety: (a) tetrahydropyran derivatives; (b) cyclohexane derivatives; (c) piperidine linkers; (d) oxabicyclooctane derivatives; (e) dioxane derivatives; and (f) piperidine-cyclobutyl linker.

Due to the spaciousness of NBTI’s binding pocket, particularly at its entrance surrounding the NBTI’s linker moiety, the linker itself makes only one critical contact with the enzyme, and not with the DNA; nevertheless, it has still an important role in the NBTIs activity.^57^ The linker is responsible for the appropriate spatial orientation of both the LHS and RHS fragments, and more importantly, it influences the physicochemical and pharmacological properties of NBTIs that are crucial for their suitable antibacterial activity and safety profile, and in particular, in their hERG inhibitory activity. The linker usually consists of three parts: an ethyl moiety connected to the LHS fragment, a cyclic moiety, and a secondary amine connected to the RHS fragment (Figures 1 and 4).^37^ The secondary amine moiety of the linker (i.e., the cationic center) has been shown to be a key structural feature for the inhibitory potency of almost all currently known NBTIs, through establishing an ionic interaction with the Asp83 residue of *S. aureus* DNA gyrase, the Asp82 residue of *E. coli* DNA gyrase, and similarly for the Asp79 residue of both *S. aureus* and *E. coli* topoIV (Figure 3a).^45^ Replacing the amine with an amide group (Figure 4e, yellow) results in a noticeably weaker enzyme inhibition, as exemplified by compounds **28** and **29** (Table S1).^45,46^

The majority of alterations to the linker have been performed on the ring and the ethyl moiety that binds to the LHS fragment. As depicted in Figure 4, the linker can contain numerous different ring moieties, including cyclohexane, 1,3-dioxane, piperidine, tetrahydropyran, oxabicyclooctane, and piperidine carboxylic acid.^22,42−44,48,51,52^

NBTIs that have fluorine and/or amino substituents at the bridgehead of the cyclohexyl moiety (Figure 4b, green and purple, respectively) generally show similar potencies against *E. coli* topoIV (Table S1, **30** and **31**).^52^ 3-Fluoro-substituted piperidines (Figure 4c, red) have shown weaker potency against *S. aureus* and *E. coli* topoIV, compared to the unsubstituted piperidines. The lower potency of these NBTI analogs is most likely due to the conformational effects of these substituents on the piperidine moiety, thereby affecting the position not only of the LHS and RHS fragments but also the spatial position of the basic amine. The piperidine moiety itself is positioned in the target-binding site in the solvent-exposed space.^37,51^ Replacement of the cyclohexane ring with tetrahydropyran decreases the NBTI potencies against both *S. aureus* and *E. coli* topoIV.^37^

The second important part of the linker is the bridge connected to the LHS fragment, and the following variants are some of the most commonly used: ethylene, oxymethylene, 1- or 2-hydroxyethylene, 1,2-dihydroxyethylene, alkene, carboxamide, and 1- or 2-aminoethylene (Figure 4a, blue).^37,43,49,51,52,59^ Hydroxyl-substituted linkers provide superior aqueous solubility of the resulting NBTIs compared to ether- and amide-containing derivatives, which also lead to stronger enzyme inhibition. NBTI analogs that contain ethylene, hydroxy-, 1, 2-dihydroxyethylene, or alkene linker bridge fragments have shown similar inhibitions (Table S1, **32**–**35**).^37,40,48^ Saturated linkers (e.g., ethylene, hydroxy, 1,2-dihydroxyethylene) allow the correct spatial geometry of the compounds, while amides, ethers, and alkenes lead to suboptimal spatial geometry of the secondary amine of the linker. However, this trend does not apply to inhibition of topoIV.^37^

It was also observed that the stereochemistry of the substituted ethylene linker affects the inhibitory activity against *E. coli* topoIV. Here, the *S*-isomer showed greater potency compared to the corresponding *R*-isomer (Table S1, **36** and **37**).^49,52^

## The Right-Hand Side Moiety

The RHS binds to the hydrophobic binding pocket of topoIV ParC subunit (Figure 1a) in a similar fashion as the targeting of the GyrA subunit of DNA gyrase. With the intention of achieving stronger binding of NBTIs to GyrA/ParC key amino acid residues, a variety of building blocks have been introduced as RHS fragments over the last two decades.

It is broadly accepted that NBTIs act as dual-targeting bacterial topoisomerase inhibitors, which most likely have less potential to develop bacterial resistance relative to those antibacterials that selectively inhibit only one of the enzymes.^60^ The high structural resemblance between amino acid residues that delineate the DNA gyrase GyrA and topoIV ParC binding pockets in Gram-positive (e.g., *S. aureus*) and Gram-negative (e.g., *E. coli*) bacterial pathogens is presumably a key aspect that governs the NBTIs dual-targeting mechanism of action.^60^ The comparison of amino acid sequences outlining the NBTIs binding pocket in GyrA and ParC subunit in *S. aureus* and *E. coli*, respectively, revealed a high structural conservation of the key amino acid residues interacting with the NBTIs (i.e., Ala68, Gly72, and Met121 in *S. aureus* GyrA; Ala67, Gly71, and Met120 in *E. coli* GyrA; Ala64, Gly68, and Met117 in *S. aureus* ParC; Ala64, Gly68, and Met118 in *E. coli* ParC) (Figure 3b). Met75 is also an important amino acid residue for NBTIs binding in *S. aureus* GyrA, which corresponds to Ile74 in *E. coli* GyrA. Ile71 is at the same position in *S. aureus* ParC, whereas in *E. coli* ParC this is Leu71^21^ (Figure S2). These latter amino acid residues are probably responsible for the slightly different potencies of the NBTIs on DNA gyrase and topoIV in Gram-positive and Gram-negative bacterial pathogens. However, due to the highly conserved binding pocket, NBTIs are sensitive to similar changes in the LHS, linker, and RHS moieties, and the overall binding of the compounds by the two targets is likely similar.

One of the first NBTIs was NXL-101, which includes a thiophene RHS moiety (Table S1, **38**).^19^ However, comprehensive research on this field suggested that a phenyl ring might be a more suitable isosteric replacement of the thiophene, as it shows less likelihood for bioactivation and, more importantly, it is a relatively straightforward substitution. To avoid any potential for oxidative metabolism, the exocyclic sulfur was removed or substituted with a carbon atom.^42^

**Figure 5:**
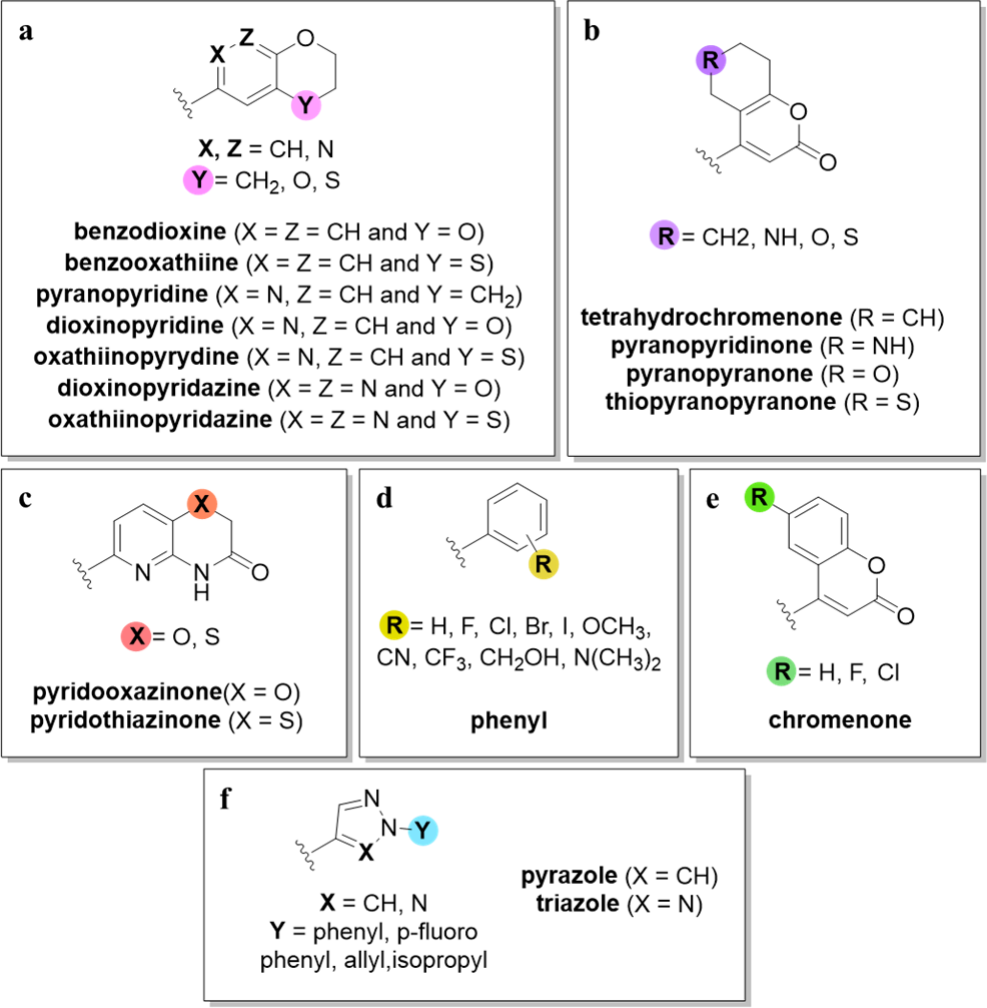
SAR guidelines of the RHS moiety: (a–c) differently substituted bicyclic derivatives; (d, f) differently substituted monocyclic derivatives; and (e) chromenone derivatives.

The most commonly used RHS moieties have been pyridooxazinone/pyridothiazinone and dioxinopyrid(az)ine/oxathiino-pyrid(az)ine (Figure 5).^37,40,43−45,48,49,51−53^ These heteroaromatic RHS fragments can establish van der Waals interactions with the surrounding hydrophobic amino acid residues that delineate the NBTI’s binding pocket of the topoIV ParC subunits.^37^

The replacement of pyridine with pyridazine reduced the inhibitory potency against topoIV (Table S1, **8** and **39**). Instead, replacement with a phenyl moiety significantly improved the enzyme inhibition (Table S1, **10** and **40**). Replacing the dioxane with tetrahydropyran or oxathiane improved the inhibitory potency (Figure 5a, pink). Compared to oxygen-containing RHS fragments, the analogs with sulfur at the same position (Figure 5a, pink) show equally potent or even stronger inhibitory potencies against topoIV (Table S1, **39/41** and **42**/**43**).^37,40,48^ Moreover, NBTIs that contain a pyridothiazinone RHS fragment consistently show stronger enzyme inhibition relative to their pyridooxazinone variants. Pyridooxazinone (or pyridothiazinone) NBTIs showed 4–16 times stronger inhibitory potencies compared to dioxinopyridine (or oxathiinopyridine) analogs (Table S1, **19** and **44**).^37,40,48^

The addition of a basic center to a cyclohexyl ring (Figure 5b, purple) reduces the inhibitory potency against *S. aureus* topoIV. Replacing cyclohexyl with tetrahydro(thio)pyran or adding an electron-withdrawing group improves the inhibition. In contrast, replacing cyclohexyl with tetrahydropyran impairs the inhibition, whereas replacing cyclohexyl with tetrahydrothiopyran or piperidine does not affect inhibition of *E. coli* topoIV (Figure 5b, purple).^44^

**Figure 6:**
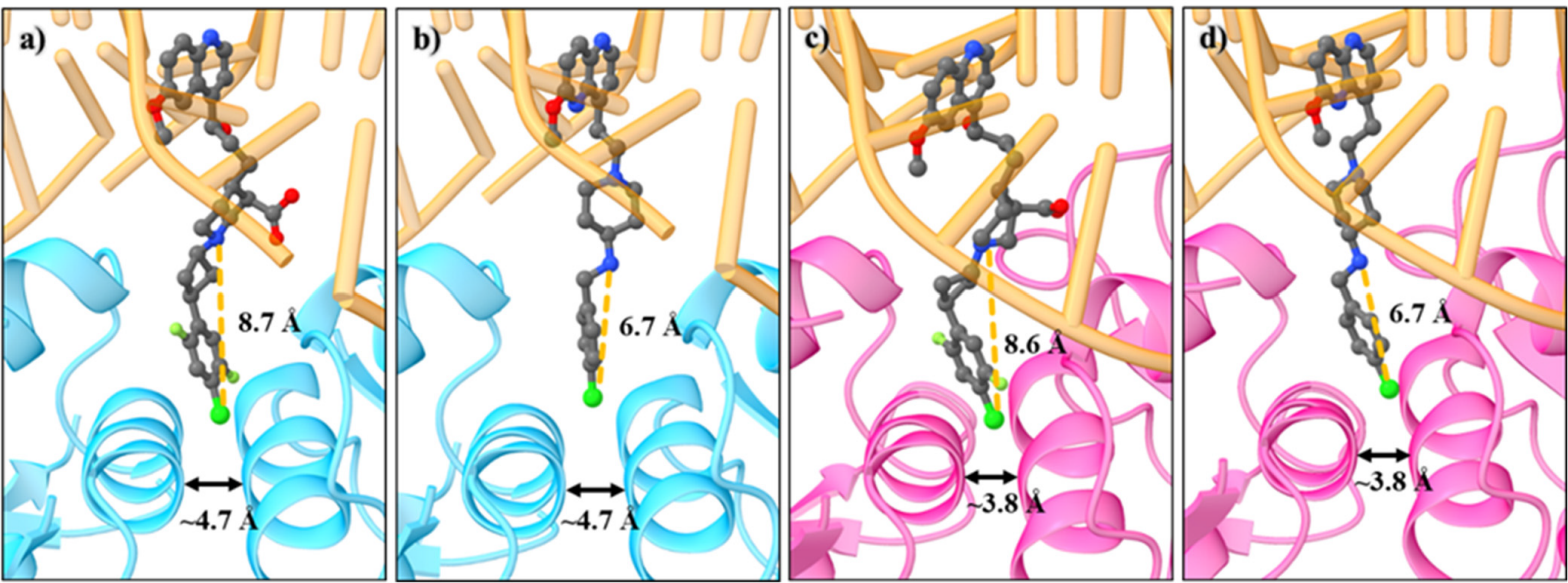
Comparisons of the binding pocket sizes of *S. aureus* DNA gyrase and topoIV as well as docking-derived poses of compounds **45** and **46**, with different RHS fragment lengths. (a) *S. aureus* DNA gyrase crystal structure (cyan, PDB ID: 6Z1A)^22^ with docked pose of **45**; (b) *S. aureus* DNA gyrase crystal structure with docked pose of **46**; (c) *S. aureus* topoIV homology model (pink) with docked pose of **45**; and (d) *S. aureus* topoIV homology model with docked pose of **46**. The enzymes are represented as cartoons, and compounds as balls and sticks, while DNA is in orange.

Various monoaromatic RHS moieties have also been studied (Figure 5d,f). One of these was the cyclobutylphenyl moiety. Substitution at the 4-aryl position with larger groups than fluorine was not favorable (e.g., −OCH_3_, −CH_3_, biphenyl, −CF_3_), and cyano substitution was suboptimal, which resulted in decreased topoIV inhibition by the phenyl analog.^42^ The reason for this might be the mere size of the ParC binding pocket in *S. aureus* topoIV compared to the GyrA binding pocket, which might result in steric hindrance of the entire RHS fragment and the poor overall inhibitory potency of the NBTIs. A comparison of the hydrophobic binding pockets between DNA gyrase and topoIV of *S. aureus* revealed that the topoIV binding pocket is a little narrower (e.g., the distance between Cα-Cα atoms of ParC α3 helices was ∼3.8 Å; Figure 6c,d), relative to that of DNA gyrase (e.g., the distance between the Cα-Cα atoms of GyrA α3 helices was ∼4.7 Å; Figure 6a,b). Fluorine substitution shows improved inhibitory activity in some cases; however, this mainly depends on the positioning of the fluorine atom on the phenyl ring. Substitutions at the *meta* position usually result in retained or improved potency. On the other hand, substitutions at the *ortho* position commonly result in improved potency, but only when combined with substitutions at the *meta* position. Among the NBTIs with difluorinated phenyl RHS, 2,5-difluorophenyl constructs showed remarkable improvements over the analogs with unsubstituted phenyl RHS.^42,36^ Nevertheless, the 2,3,5-trifluorophenyl analog showed the strongest topoIV inhibition in this studied NBTI series.^36^

For NBTIs with monoaromatic RHS, compounds with halogens at the *para* position on the phenyl RHS fragment have also been studied in correlation with their topoIV inhibitory potencies. In one case, increasing the size of the halogen atom led to reduced potency. The reason for this might lie in the length of the RHS fragment (e.g., the distance between the linker amine and the most distant RHS atom is ∼8.7 Å in GyrA and ∼8.6 Å in ParC; compound **45**, Table S1 and Figure 6a,c), which in turn might prevent the formation of halogen-bonding interactions between the *p*-halogen atom and the backbone carbonyl oxygens of the Ala64 residue in *S. aureus* ParC. In our previous studies,^22,38^ we demonstrated that the inhibitory potencies of NBTIs with *p*-halogenated phenyl RHS fragments against topoIV increased down the halogen group of the periodic table of elements (Table S1, **46**–**48**). The reason for this is most likely the formation of halogen-bonding interactions between the *p*-halogen atom and the backbone carbonyl oxygens of Ala64 ParC in the *S. aureus* and/or *E. coli* GyrA in a similar fashion as was observed in the crystal structure of *S. aureus* DNA gyrase in complex with DNA and the AMK-12 ligand (PDB ID: 6Z1A).^22^ Here, the length of the RHS fragment was substantially shorter (e.g., the distance between the linker amine and the most distant RHS atom is ∼6.7 Å; compound **46**, Table S1 and Figure 6b,d). This result clearly indicates the potential to establish halogen-bonding interactions that lead to strong inhibitory activities of this series of NBTIs. As a continuation of this work, we also demonstrated that the inhibitory potencies of NBTIs with *p*-halogenated phenyl RHS fragments can be enhanced by increasing their electron-withdrawing properties as well as halogen-bonding propensities, while maintaining the same lipophilicity and approximately the same RHS size (Table S1, **47**, **49**, and **50**).^41^ Put differently, the flexibility provided by replacement of a bicyclic heteroaromatic RHS moiety with a monocyclic one (e.g., a halogenated phenyl) and its suitable length are crucial to establish halogen-bonding interactions with the Ala residues and result in a stronger enzyme inhibition.^38^ Moreover, the replacement of triazole with pyrazole (Figure 5f, blue) generally leads to increased inhibitory potency. Replacing phenyl with isopropyl does not have any effects on the inhibitory potency against either *S. aureus* or *E. coli*, although relative to phenyl, propene decreases the inhibition of topoIV in both of these bacteria.^38^

As it is the RHS that interacts solely with the enzymes, it should be regarded as a major structural feature that directly discriminates the selectivity between DNA gyrase and topoIV. It should be stressed, however, that although LHS and linker moieties do not have direct impact on the enzyme inhibition, they significantly contribute to the proper spatial positioning of the RHS fragment within the hydrophobic binding pocket of the enzymes and the overall, suitable physicochemical properties of NBTIs, as well.^52^

## Conclusion

In this perspective, we have summarized the structural features of each of the parts (i.e., LHS, linker, RHS) of the antibacterials from the NBTI class that govern topoIV inhibitory activity. Each part of NBTIs individually affects topoIV inhibition. The LHS with its intercalation into the DNA base pairs, the linker through the geometrical positioning of the LHS and RHS fragments, and the RHS moiety with its binding into the enzyme pocket. Considering the high structural similarity that DNA gyrase and topoIV share, particularly in the NBTI binding pocket, dual inhibition of both enzymes with a single compound is reasonable to expect. Introduction of small fragments, such as fluorine and methoxy groups in the LHS fragments, provided favorable effects against both bacterial type II topoisomerases. In both cases, the linker moiety is responsible not only for providing suitable physiochemical properties of the entire ligand but also for the correct geometric positioning of the LHS and RHS fragments, respectively. Moreover, the protonated basic amine of the linker moiety has been shown to be optimal for NBTIs activity. As the RHS fragment interacts with both enzymes, it should be regarded as a major structural feature that discriminates between DNA gyrase and topoIV NBTIs affinity. Since, the dual inhibition lowers the possibility of developing bacterial resistance, the design of NBTIs should rely on targeting both enzymes. With improved enzyme inhibition against topoIV, an improved dual inhibition can be achieved especially in Gram-negative bacteria, where topoIV is most probably a primary target of NBTIs. Unfortunately, no structural data of topoIV in complex with DNA and an NBTI inhibitor have been published to date. Hence, the next key objective of the scientific community would be the revelation of an atomic-resolution structure of the topoIV enzyme in complex with an intercalated NBTI ligand, which in turn will enable a more intuitive structure-based design. This, along with some other hurdles need to be overcome prior to defining NBTIs as safe enough and suitably active to slow down the rapidly spread of resistance to existing antibacterials. To facilitate these efforts, we present here some general guidelines toward improving NBTIs inhibition of topoIV.

## Supporting Information Available

A table of NBTI representatives targeting topoIV enzyme, a superposition of cocrystallized NBTI ligands from the available X-ray DNA gyrase/DNA/NBTI complexes as well as a comparison of amino acid sequences delineating NBTIs binding pocket of GyrA and ParC subunits in DNA gyrase and topoIV, respectively from various Gram-positive and Gram-negative bacterial pathogens (PDF)

## Abbreviations Used

LHS: left-hand side
NBTIs: novel bacterial topoisomerase inhibitors
RHS: right-hand side
SARs: structure–activity relationships
topoIV: topoisomerase IV

## References

[ref1] Chambers H. F.; DeLeo F. R. Waves of Resistance: Staphylococcus Aureus in the Antibiotic Era. Nat. Rev. Microbiol. 2009, 7 (9), 629–641. 10.1038/nrmicro2200. 19680247 PMC2871281

[ref2] Centers for Disease Control and Prevention. https://www.cdc.gov/drugresistance/pdf/threats-report/2019-ar-threats-report-508.pdf (accessed Oct 5, 2021).

[ref3] Silver L. L. A Gestalt Approach to Gram-Negative Entry. Bioorg. Med. Chem. 2016, 24 (24), 6379–6389. 10.1016/j.bmc.2016.06.044. 27381365

[ref4] De Oliveira D. M. P.; Forde B. M.; Kidd T. J.; Harris P. N. A.; Schembri M. A.; Beatson S. A.; Paterson D. L.; Walker M. J. Antimicrobial Resistance in ESKAPE Pathogens. Clin. Microbiol. Rev. 2020, 33 (3), 1–49. 10.1128/CMR.00181-19. PMC7227449 32404435

[ref5] Rice L. B. Federal Funding for the Study of Antimicrobial Resistance in Nosocomial Pathogens: No ESKAPE. J. Infect. Dis. 2008, 197 (8), 1079–1081. 10.1086/533452. 18419525

[ref6] Murray C. J.; Ikuta K. S.; Sharara F.; Swetschinski L.; Robles Aguilar G.; Gray A.; Han C.; Bisignano C.; Rao P.; Wool E.; Johnson S. C.; Browne A. J.; Chipeta M. G.; Fell F.; Hackett S.; Haines-Woodhouse G.; Kashef Hamadani B. H.; Kumaran E. A. P.; McManigal B.; Agarwal R.; Akech S.; Albertson S.; Amuasi J.; Andrews J.; Aravkin A.; Ashley E.; Bailey F.; Baker S.; Basnyat B.; Bekker A.; Bender R.; Bethou A.; Bielicki J.; Boonkasidecha S.; Bukosia J.; Carvalheiro C.; Castañeda-Orjuela C.; Chansamouth V.; Chaurasia S.; Chiurchiù S.; Chowdhury F.; Cook A. J.; Cooper B.; Cressey T. R.; Criollo-Mora E.; Cunningham M.; Darboe S.; Day N. P. J.; De Luca M.; Dokova K.; Dramowski A.; Dunachie S. J.; Eckmanns T.; Eibach D.; Emami A.; Feasey N.; Fisher-Pearson N.; Forrest K.; Garrett D.; Gastmeier P.; Giref A. Z.; Greer R. C.; Gupta V.; Haller S.; Haselbeck A.; Hay S. I.; Holm M.; Hopkins S.; Iregbu K. C.; Jacobs J.; Jarovsky D.; Javanmardi F.; Khorana M.; Kissoon N.; Kobeissi E.; Kostyanev T.; Krapp F.; Krumkamp R.; Kumar A.; Kyu H. H.; Lim C.; Limmathurotsakul D.; Loftus M. J.; Lunn M.; Ma J.; Mturi N.; Munera-Huertas T.; Musicha P.; Mussi-Pinhata M. M.; Nakamura T.; Nanavati R.; Nangia S.; Newton P.; Ngoun C.; Novotney A.; Nwakanma D.; Obiero C. W.; Olivas-Martinez A.; Olliaro P.; Ooko E.; Ortiz-Brizuela E.; Peleg A. Y.; Perrone C.; Plakkal N.; Ponce-de-Leon A.; Raad M.; Ramdin T.; Riddell A.; Roberts T.; Robotham J. V.; Roca A.; Rudd K. E.; Russell N.; Schnall J.; Scott J. A. G.; Shivamallappa M.; Sifuentes-Osornio J.; Steenkeste N.; Stewardson A. J.; Stoeva T.; Tasak N.; Thaiprakong A.; Thwaites G.; Turner C.; Turner P.; van Doorn H. R.; Velaphi S.; Vongpradith A.; Vu H.; Walsh T.; Waner S.; Wangrangsimakul T.; Wozniak T.; Zheng P.; Sartorius B.; Lopez A. D.; Stergachis A.; Moore C.; Dolecek C.; Naghavi M. Global Burden of Bacterial Antimicrobial Resistance in 2019: A Systematic Analysis. Lancet 2022, 399 (10325), 629–655. 10.1016/S0140-6736(21)02724-0. 35065702 PMC8841637

[ref7] Review on Antimicrobial Resistance: Tackling a crisis for the health and wealth of nations. https://amr-review.org/sites/default/files/AMR%20Review%20Paper%20-%20Tackling%20a%20crisis%20for%20the%20health%20and%20wealth%20of%20nations_1.pdf (accessed 2021-11-05).

[ref8] Khan T.; Sankhe K.; Suvarna V.; Sherje A.; Patel K.; Dravyakar B. DNA Gyrase Inhibitors: Progress and Synthesis of Potent Compounds as Antibacterial Agents. Biomed. Pharmacother. 2018, 103, 923–938. 10.1016/j.biopha.2018.04.021. 29710509

[ref9] Bansal S.; Bajaj P.; Pandey S.; Tandon V. Topoisomerases: Resistance versus Sensitivity, How FarWe Can Go?. Med. Res. Rev. 2017, 37 (2), 404–438. 10.1002/med.21417. 27687257

[ref10] Bush N. G.; Evans-Roberts K.; Maxwell A. DNA Topoisomerases. EcoSal Plus 2015, 6, 1–34. 10.1128/ecosalplus.ESP-0010-2014. PMC11575854 26435256

[ref11] van Eijk E.; Wittekoek B.; Kuijper E. J.; Smits W. K. DNA Replication Proteins as Potential Targets for Antimicrobials in Drug-Resistant Bacterial Pathogens. J. Antimicrob. Chemother. 2017, 72 (5), 1275–1284. 10.1093/jac/dkw548. 28073967 PMC5400081

[ref12] Gibson E. G.; Bax B.; Chan P. F.; Osheroff N. Mechanistic and Structural Basis for the Actions of the Antibacterial Gepotidacin against Staphylococcus Aureus Gyrase. ACS Infect. Dis. 2019, 5 (4), 570–581. 10.1021/acsinfecdis.8b00315. 30757898 PMC6461504

[ref13] Hiasa H. DNA Topoisomerases as Targets for Antibacterial Agents. Methods Mol. Biol. 2018, 1703, 47–62. 10.1007/978-1-4939-7459-7_3. 29177732

[ref14] Piton J.; Petrella S.; Delarue M.; André-Leroux G.; Jarlier V.; Aubry A.; Mayer C. Structural Insights into the Quinolone Resistance Mechanism of Mycobacterium Tuberculosis DNA Gyrase. PLoS One 2010, 5 (8), e12245 10.1371/journal.pone.0012245. 20805881 PMC2923608

[ref15] Martínez J. L. Mechanisms of Action and of Resistance to Quinolones. Antibiotic Drug Resistance; Wiley: Hoboken, NJ, 2019. Vol. 01805, (Suppl 2), , pp 39–55.

[ref16] Miles T. J.; Axten J. M.; Barfoot C.; Brooks G.; Brown P.; Chen D.; Dabbs S.; Davies D. T.; Downie D. L.; Eyrisch S.; Gallagher T.; Giordano I.; Gwynn M. N.; Hennessy A.; Hoover J.; Huang J.; Jones G.; Markwell R.; Miller W. H.; Minthorn E. A.; Rittenhouse S.; Seefeld M.; Pearson N. Novel Amino-Piperidines as Potent Antibacterials Targeting Bacterial Type IIA Topoisomerases. Bioorg. Med. Chem. Lett. 2011, 21 (24), 7489–7495. 10.1016/j.bmcl.2011.09.117. 22047689

[ref17] Wiener J. J. M.; Gomez L.; Venkatesan H.; Santillán A.; Allison B. D.; Schwarz K. L.; Shinde S.; Tang L.; Hack M. D.; Morrow B. J.; Motley S. T.; Goldschmidt R. M.; Shaw K. J.; Jones T. K.; Grice C. A. Tetrahydroindazole Inhibitors of Bacterial Type II Topoisomerases. Part 2: SAR Development and Potency against Multidrug-Resistant Strains. Bioorg. Med. Chem. Lett. 2007, 17 (10), 2718–2722. 10.1016/j.bmcl.2007.03.004. 17382544

[ref18] Gomez L.; Hack M. D.; Wu J.; Wiener J. J. M.; Venkatesan H.; Santillán A.; Pippel D. J.; Mani N.; Morrow B. J.; Motley S. T.; Shaw K. J.; Wolin R.; Grice C. A.; Jones T. K. Novel Pyrazole Derivatives as Potent Inhibitors of Type II Topoisomerases. Part 1: Synthesis and Preliminary SAR Analysis. Bioorg. Med. Chem. Lett. 2007, 17 (10), 2723–2727. 10.1016/j.bmcl.2007.03.003. 17368897

[ref19] Black M. T.; Stachyra T.; Platel D.; Girard A. M.; Claudon M.; Bruneau J. M.; Miossec C. Mechanism of Action of the Antibiotic NXL101, a Novel Nonfluoroquinolone Inhibitor of Bacterial Type II Topoisomerases. Antimicrob. Agents Chemother. 2008, 52 (9), 3339–3349. 10.1128/AAC.00496-08. 18625781 PMC2533460

[ref20] Bax B. D.; Chan P. F.; Eggleston D. S.; Fosberry A.; Gentry D. R.; Gorrec F.; Giordano I.; Hann M. M.; Hennessy A.; Hibbs M.; Huang J.; Jones E.; Jones J.; Brown K. K.; Lewis C. J.; May E. W.; Saunders M. R.; Singh O.; Spitzfaden C. E.; Shen C.; Shillings A.; Theobald A. J.; Wohlkonig A.; Pearson N. D.; Gwynn M. N. Type IIA Topoisomerase Inhibition by a New Class of Antibacterial Agents. Nature 2010, 466 (7309), 935–940. 10.1038/nature09197. 20686482

[ref21] Kolarič A.; Anderluh M.; Minovski N. Two Decades of Successful SAR-Grounded Stories of the Novel Bacterial Topoisomerase Inhibitors (NBTIs). J. Med. Chem. 2020, 63 (11), 5664–5674. 10.1021/acs.jmedchem.9b01738. 32027491 PMC7307926

[ref22] Kolarič A.; Germe T.; Hrast M.; Stevenson C. E. M.; Lawson D. M.; Burton N. P.; Vörös J.; Maxwell A.; Minovski N.; Anderluh M. Potent DNA Gyrase Inhibitors Bind Asymmetrically to Their Target Using Symmetrical Bifurcated Halogen Bonds. Nat. Commun. 2021, 12 (1), 150 10.1038/s41467-020-20405-8. 33420011 PMC7794245

[ref23] Ehmann D. E.; Lahiri S. D. Novel Compounds Targeting Bacterial DNA Topoisomerase/DNA Gyrase. Curr. Opin. Pharmacol. 2014, 18, 76–83. 10.1016/j.coph.2014.09.007. 25271174

[ref24] Tse-Dinh Y.-C. Targeting Bacterial Topoisomerases - How to Counter Mechanisms of Resistance. Futur. Med. Chem. 2016, 8 (10), 1085–1100. 10.4155/fmc-2016-0042. 27285067

[ref25] Miller A. A.; Bundy G. L.; Mott J. E.; Skepner J. E.; Boyle T. P.; Harris D. W.; Hromockyj A. E.; Marotti K. R.; Zurenko G. E.; Munzner J. B.; Sweeney M. T.; Bammert G. F.; Hamel J. C.; Ford C. W.; Zhong W. Z.; Graber D. R.; Martin G. E.; Han F.; Dolak L. A.; Seest E. P.; Ruble J. C.; Kamilar G. M.; Palmer J. R.; Banitt L. S.; Hurd A. R.; Barbachyn M. R. Discovery and Characterization of QPT-1, the Progenitor of a New Class of Bacterial Topoisomerase Inhibitors. Antimicrob. Agents Chemother. 2008, 52 (8), 2806–2812. 10.1128/AAC.00247-08. 18519725 PMC2493097

[ref26] Bradford P. A.; Miller A. A.; O’Donnell J.; Mueller J. P. Zoliflodacin: An Oral Spiropyrimidinetrione Antibiotic for the Treatment of Neisseria Gonorrheae, Including Multi-Drug-Resistant Isolates. ACS Infect. Dis. 2020, 6 (6), 1332–1345. 10.1021/acsinfecdis.0c00021. 32329999

[ref27] Black M. T.; Coleman K. New Inhibitors of Bacterial Topoisomerase GyrA/ParC Subunits. Curr. Opin. Investig. Drugs 2009, 10 (8), 804–810. 19649925

[ref28] Scangarella-Oman N. E.; Hossain M.; Dixon P. B.; Ingraham K.; Min S.; Tiffany C. A.; Perry C. R.; Raychaudhuri A.; Dumont E. F.; Huang J.; Hook E. W.; Miller L. A. Microbiological Analysis from a Phase 2 Randomized Study in Adults Evaluating Single Oral Doses of Gepotidacin in the Treatment of Uncomplicated Urogenital Gonorrhea Caused by Neisseria Gonorrhoeae. Antimicrob. Agents Chemother. 2018, 62 (12), e01221 10.1128/AAC.01221-18. 30249694 PMC6256812

[ref29] VanScoy B. D.; Scangarella-Oman N. E.; Fikes S.; Min S.; Huang J.; Ingraham K.; Bhavnani S. M.; Conde H.; Ambrose P. G. Relationship between Gepotidacin Exposure and Prevention of On-Therapy Resistance Amplification in a Neisseria Gonorrhoeae Hollow-Fiber In Vitro Infection Model. Antimicrob. Agents Chemother. 2020, 64 (10), e00521 10.1128/AAC.00521-20. 32661002 PMC7508576

[ref30] Nuzzo A.; Van Horn S.; Traini C.; Perry C. R.; Dumont E. F.; Scangarella-Oman N. E.; Gardiner D. F.; Brown J. R. Microbiome Recovery in Adult Females with Uncomplicated Urinary Tract Infections in a Randomised Phase 2A Trial of the Novel Antibiotic Gepotidacin (GSK2140944). BMC Microbiol. 2021, 21 (1), 181 10.1186/s12866-021-02245-8. 34130619 PMC8207760

[ref31] Scangarella-Oman N. E.; Hossain M.; Hoover J. L.; Perry C. R.; Tiffany C.; Barth A.; Dumont E. F. Dose Selection for Phase III Clinical Evaluation of Gepotidacin (GSK2140944) in the Treatment of Uncomplicated Urinary Tract Infections. Antimicrob. Agents Chemother. 2022, 66 (3), 01492 10.1128/aac.01492-21. PMC8923173 34978887

[ref32] Chan P.; Ingraham K.; Min S.; Scangarella- Oman N.; Rittenhouse S.; Huang J. Genetic Evidence That Gepotidacin Shows Well-Balanced Dual Targeting against DNA Gyrase And Topoisomerase IV in Neisseria Gonorrhoeae. OFID, Poster Abstr. 2020, 7, S642–S643. 10.1093/ofid/ofaa439.1433.

[ref33] Scangarella-Oman N. E.; Ingraham K. A.; Tiffany C. A.; Tomsho L.; Van Horn S. F.; Mayhew D. N.; Perry C. R.; Ashton T. C.; Dumont E. F.; Huang J.; Brown J. R.; Miller L. A. In Vitro Activity and Microbiological Efficacy of Gepotidacin from a Phase 2, Randomized, Multicenter, Dose-Ranging Study in Patients with Acute Bacterial Skin and Skin Structure Infections. Antimicrob Agents Chemother 2020, 64 (3), e01302 10.1128/AAC.01302-19. 31818823 PMC7038298

[ref34] Singh S. B.; Kaelin D. E.; Wu J.; Miesel L.; Tan C. M.; Meinke P. T.; Olsen D. B.; Lagrutta A.; Wei C.; Liao Y.; Peng X.; Wang X.; Fukuda H.; Kishii R.; Takei M.; Shibata T.; Takeuchi T.; Ohata K.; Nishimura A.; Fukuda Y. C1-C2-Linker Substituted 1,5-Naphthyridine Analogues of Oxabicyclooctane-Linked NBTIs as Broad-Spectrum Antibacterial Agents (Part 7). Medchemcomm 2015, 6 (10), 1773–1780. 10.1039/C5MD00297D.

[ref35] Badshah S. L.; Ullah A. New Developments in Non-Quinolone-Based Antibiotics for the Inhibiton of Bacterial Gyrase and Topoisomerase IV. Eur. J. Med. Chem. 2018, 152, 393–400. 10.1016/j.ejmech.2018.04.059. 29751233

[ref36] Mitton-Fry M. J.; Brickner S. J.; Hamel J. C.; Barham R.; Brennan L.; Casavant J. M.; Ding X.; Finegan S.; Hardink J.; Hoang T.; Huband M. D.; Maloney M.; Marfat A.; McCurdy S. P.; McLeod D.; Subramanyam C.; Plotkin M.; Reilly U.; Schafer J.; Stone G. G.; Uccello D. P.; Wisialowski T.; Yoon K.; Zaniewski R.; Zook C. Novel 3-Fluoro-6-Methoxyquinoline Derivatives as Inhibitors of Bacterial DNA Gyrase and Topoisomerase IV. Bioorg. Med. Chem. Lett. 2017, 27 (15), 3353–3358. 10.1016/j.bmcl.2017.06.009. 28610977

[ref37] Surivet J. P.; Zumbrunn C.; Rueedi G.; Hubschwerlen C.; Bur D.; Bruyère T.; Locher H.; Ritz D.; Keck W.; Seiler P.; Kohl C.; Gauvin J. C.; Mirre A.; Kaegi V.; Dos Santos M.; Gaertner M.; Delers J.; Enderlin-Paput M.; Boehme M. Design, Synthesis, and Characterization of Novel Tetrahydropyran-Based Bacterial Topoisomerase Inhibitors with Potent Anti-Gram-Positive Activity. J. Med. Chem. 2013, 56 (18), 7396–7415. 10.1021/jm400963y. 23968485

[ref38] Kolarič A.; Kokot M.; Hrast M.; Weiss M.; Zdovc I.; Trontelj J.; Žakelj S.; Anderluh M.; Minovski N. A Fine-Tuned Lipophilicity/Hydrophilicity Ratio Governs Antibacterial Potency and Selectivity of Bifurcated Halogen. Antibiotics 2021, 10 (7), 862 10.3390/antibiotics10070862. 34356782 PMC8300687

[ref39] Nayar A. S.; Dougherty T. J.; Reck F.; Thresher J.; Gao N.; Shapiro A. B.; Ehmann D. E. Target-Based Resistance in Pseudomonas Aeruginosa and Escherichia Coli to NBTI 5463, a Novel Bacterial Type II Topoisomerase Inhibitor. Antimicrob. Agents Chemother. 2015, 59 (1), 331–337. 10.1128/AAC.04077-14. 25348539 PMC4291369

[ref40] Lu Y.; Vibhute S.; Li L.; Okumu A.; Ratigan S. C.; Nolan S.; Papa J. L.; Mann C. A.; English A.; Chen A.; Seffernick J. T.; Koci B.; Duncan L. R.; Roth B.; Cummings J. E.; Slayden R. A.; Lindert S.; McElroy C. A.; Wozniak D. J.; Yalowich J.; Mitton-Fry M. J. Optimization of TopoIV Potency, ADMET Properties, and HERG Inhibition of 5-Amino-1,3-Dioxane-Linked Novel Bacterial Topoisomerase Inhibitors: Identification of a Lead with in Vivo Efficacy against MRSA. J. Med. Chem. 2021, 64 (20), 15214–15249. 10.1021/acs.jmedchem.1c01250. 34614347

[ref41] Kokot M.; Weiss M.; Zdovc I.; Hrast M.; Anderluh M.; Minovski N. Structurally Optimized Potent Dual-Targeting NBTI Antibacterials with an Enhanced Bifurcated Halogen-Bonding Propensity. ACS Med. Chem. Lett. 2021, 12 (9), 1478–1485. 10.1021/acsmedchemlett.1c00345. 34527181 PMC8436411

[ref42] Mitton-Fry M. J.; Brickner S. J.; Hamel J. C.; Brennan L.; Casavant J. M.; Chen M.; Chen T.; Ding X.; Driscoll J.; Hardink J.; Hoang T.; Hua E.; Huband M. D.; Maloney M.; Marfat A.; McCurdy S. P.; McLeod D.; Plotkin M.; Reilly U.; Robinson S.; Schafer J.; Shepard R. M.; Smith J. F.; Stone G. G.; Subramanyam C.; Yoon K.; Yuan W.; Zaniewski R. P.; Zook C. Novel Quinoline Derivatives as Inhibitors of Bacterial DNA Gyrase and Topoisomerase IV. Bioorg. Med. Chem. Lett. 2013, 23 (10), 2955–2961. 10.1016/j.bmcl.2013.03.047. 23566517

[ref43] Li L.; Okumu A. A.; Nolan S.; English A.; Vibhute S.; Lu Y.; Hervert-Thomas K.; Seffernick J. T.; Azap L.; Cole S. L.; Shinabarger D.; Koeth L. M.; Lindert S.; Yalowich J. C.; Wozniak D. J.; Mitton-Fry M. J. 1,3-Dioxane-Linked Bacterial Topoisomerase Inhibitors with Enhanced Antibacterial Activity and Reduced HERG Inhibition. ACS Infect. Dis. 2019, 5 (7), 1115–1128. 10.1021/acsinfecdis.8b00375. 31041863

[ref44] Magarò G.; Prati F.; Garofalo B.; Corso G.; Furlotti G.; Apicella C.; Mangano G.; D’Atanasio N.; Robinson D.; Di Giorgio F. P.; Ombrato R. Virtual Screening Approach and Investigation of Structure-Activity Relationships to Discover Novel Bacterial Topoisomerase Inhibitors Targeting Gram-Positive and Gram-Negative Pathogens. J. Med. Chem. 2019, 62 (16), 7445–7472. 10.1021/acs.jmedchem.9b00394. 31276392

[ref45] Li L.; Okumu A.; Dellos-Nolan S.; Li Z.; Karmahapatra S.; English A.; Yalowich J. C.; Wozniak D. J.; Mitton-Fry M. J. Synthesis and Anti-Staphylococcal Activity of Novel Bacterial Topoisomerase Inhibitors with a 5-Amino-1,3-Dioxane Linker Moiety. Bioorg. Med. Chem. Lett. 2018, 28 (14), 2477–2480. 10.1016/j.bmcl.2018.06.003. 29871847

[ref46] Lu Y.; Papa J. L.; Nolan S.; English A.; Seffernick J. T.; Shkolnikov N.; Powell J.; Lindert S.; Wozniak D. J.; Yalowich J.; Mitton-Fry M. J. Dioxane-Linked Amide Derivatives as Novel Bacterial Topoisomerase Inhibitors against Gram-Positive Staphylococcus Aureus. ACS Med. Chem. Lett. 2020, 11 (12), 2446–2454. 10.1021/acsmedchemlett.0c00428. 33335666 PMC7734797

[ref47] Charrier C.; Salisbury A.-M.; Savage V. J.; Duffy T.; Moyo E.; Chaffer-Malam N.; Ooi N.; Newman R.; Cheung J.; Metzger R.; McGarry D.; Pichowicz M.; Sigerson R.; Cooper I. R.; Nelson G.; Butler H. S.; Craighead M.; Ratcliffe A. J.; Best S. A.; Stokes N. R. Novel Bacterial Topoisomerase Inhibitors with Potent Broad-Spectrum Activity against Drug-Resistant Bacteria Skin and Skin Structure Infections. Antimicrob. Agents Chemother. 2017, 61 (5), 02100 10.1128/AAC.02100-16. PMC5404544 28223393

[ref48] Surivet J. P.; Zumbrunn C.; Rueedi G.; Bur D.; Bruyère T.; Locher H.; Ritz D.; Seiler P.; Kohl C.; Ertel E. A.; Hess P.; Gauvin J. C.; Mirre A.; Kaegi V.; Dos Santos M.; Kraemer S.; Gaertner M.; Delers J.; Enderlin-Paput M.; Weiss M.; Sube R.; Hadana H.; Keck W.; Hubschwerlen C. Novel Tetrahydropyran-Based Bacterial Topoisomerase Inhibitors with Potent Anti-Gram Positive Activity and Improved Safety Profile. J. Med. Chem. 2015, 58 (2), 927–942. 10.1021/jm501590q. 25494934

[ref49] Surivet J. P.; Zumbrunn C.; Bruyère T.; Bur D.; Kohl C.; Locher H. H.; Seiler P.; Ertel E. A.; Hess P.; Enderlin-Paput M.; Enderlin-Paput S.; Gauvin J. C.; Mirre A.; Hubschwerlen C.; Ritz D.; Rueedi G. Synthesis and Characterization of Tetrahydropyran-Based Bacterial Topoisomerase Inhibitors with Antibacterial Activity against Gram-Negative Bacteria. J. Med. Chem. 2017, 60 (9), 3776–3794. 10.1021/acs.jmedchem.6b01831. 28406300

[ref50] Reck F.; Alm R.; Brassil P.; Newman J.; DeJonge B.; Eyermann C. J.; Breault G.; Breen J.; Comita-Prevoir J.; Cronin M.; Davis H.; Ehmann D. E.; Galullo V.; Geng B.; Grebe T.; Morningstar M.; Walker P.; Hayter B.; Fisher S. Novel N-Linked Aminopiperidine Inhibitors of Bacterial Topoisomerase Type II: Broad-Spectrum Antibacterial Agents with Reduced HERG Activity. J. Med. Chem. 2011, 54 (22), 7834–7847. 10.1021/jm2008826. 21999508

[ref51] Reck F.; Alm R. A.; Brassil P.; Newman J. V.; Ciaccio P.; McNulty J.; Barthlow H.; Goteti K.; Breen J.; Comita-Prevoir J.; Cronin M.; Ehmann D. E.; Geng B.; Godfrey A. A.; Fisher S. L. Novel N-Linked Aminopiperidine Inhibitors of Bacterial Topoisomerase Type II with Reduced p K a: Antibacterial Agents with an Improved Safety Profile. J. Med. Chem. 2012, 55 (15), 6916–6933. 10.1021/jm300690s. 22779424

[ref52] Reck F.; Ehmann D. E.; Dougherty T. J.; Newman J. V.; Hopkins S.; Stone G.; Agrawal N.; Ciaccio P.; McNulty J.; Barthlow H.; O’Donnell J.; Goteti K.; Breen J.; Comita-Prevoir J.; Cornebise M.; Cronin M.; Eyermann C. J.; Geng B.; Carr G. R.; Pandarinathan L.; Tang X.; Cottone A.; Zhao L.; Bezdenejnih-Snyder N. Optimization of Physicochemical Properties and Safety Profile of Novel Bacterial Topoisomerase Type II Inhibitors (NBTIs) with Activity against Pseudomonas Aeruginosa. Bioorg. Med. Chem. 2014, 22 (19), 5392–5409. 10.1016/j.bmc.2014.07.040. 25155913

[ref53] Geng B.; Comita-Prevoir J.; Eyermann C. J.; Reck F.; Fisher S. Exploring Left-Hand-Side Substitutions in the Benzoxazinone Series of 4-Amino-Piperidine Bacterial Type IIa Topoisomerase Inhibitors. Bioorg. Med. Chem. Lett. 2011, 21 (18), 5432–5435. 10.1016/j.bmcl.2011.06.126. 21782427

[ref54] Gibson E. G.; Oviatt A. A.; Cacho M.; Neuman K. C.; Chan P. F.; Osheroff N. Bimodal Actions of a Naphthyridone/Aminopiperidine-Based Antibacterial That Targets Gyrase and Topoisomerase IV. Biochemistry 2019, 58 (44), 4447–4455. 10.1021/acs.biochem.9b00805. 31617352 PMC7450530

[ref55] Müller K.; Faeh C.; Diederich F. Fluorine in Pharmaceuticals: Looking beyond Intuition. Science (80-.). 2007, 317 (5846), 1881–1886. 10.1126/science.1131943. 17901324

[ref56] Tan C. M.; Gill C. J.; Wu J.; Toussaint N.; Yin J.; Tsuchiya T.; Garlisi C. G.; Kaelin D.; Meinke P. T.; Miesel L.; Olsen D. B.; Lagrutta A.; Fukuda H.; Kishii R.; Takei M.; Oohata K.; Takeuchi T.; Shibue T.; Takano H.; Nishimura A.; Fukuda Y.; Singh S. B. In Vitro and in Vivo Characterization of the Novel Oxabicyclooctane-Linked Bacterial Topoisomerase Inhibitor AM-8722, a Selective, Potent Inhibitor of Bacterial DNA Gyrase. Antimicrob. Agents Chemother. 2016, 60 (8), 4830–4839. 10.1128/AAC.00619-16. 27246784 PMC4958163

[ref57] Kolarič A.; Minovski N. Novel Bacterial Topoisomerase Inhibitors: Challenges and Perspectives in Reducing HERG Toxicity. Future Med. Chem. 2018, 10 (19), 2241–2244. 10.4155/fmc-2018-0272. 30215281

[ref58] Vanden Broeck A.; Lotz C.; Ortiz J.; Lamour V. Cryo-EM Structure of the Complete E. Coli DNA Gyrase Nucleoprotein Complex. Nat. Commun. 2019, 10 (1), 4935 10.1038/s41467-019-12914-y. 31666516 PMC6821735

[ref59] Singh S. B.; Kaelin D. E.; Wu J.; Miesel L.; Tan C. M.; Meinke P. T.; Olsen D.; Lagrutta A.; Bradley P.; Lu J.; Patel S.; Rickert K. W.; Smith R. F.; Soisson S.; Wei C.; Fukuda H.; Kishii R.; Takei M.; Fukuda Y. Oxabicyclooctane-Linked Novel Bacterial Topoisomerase Inhibitors as Broad Spectrum Antibacterial Agents. ACS Med. Chem. Lett. 2014, 5 (5), 609–614. 10.1021/ml500069w. 24900889 PMC4027601

[ref60] Tomasic T.; Masic L. P. Prospects for Developing New Antibacterials Targeting Bacterial Type IIA Topoisomerases. Curr. Top. Med. Chem. 2013, 14, 130–151. 10.2174/1568026613666131113153251. 24236722

