# Supplementary material for: The Structural Features of Novel Bacterial Topoisomerase Inhibitors That Define Their Activity on Topoisomerase IV
Source: J Med Chem. 2022 May 3;65(9):6431–40. doi: 10.1021/acs.jmedchem.2c00039 (PMC9109137; doi:10.1021/acs.jmedchem.2c00039)
Supplement: Supplementary file 1 — jm2c00039_si_001.pdf [file jm2c00039_si_001.pdf]

Supporting Information

The Structural Features of Novel Bacterial Topoisomerase Inhibitors that Define Their Activity on Topoisomerase IV

Maja Kokot [a, b], Marko Anderluh [b], Martina Hrast *[b], Nikola Minovski *[a]

*Corresponding authors. Tel. +386 1 4760 383, e-mail: nikola.minovski@ki.si, Tel: +386 1 4769 674; e-mail: martina.hrast@ffa.uni-lj.si

[a] Theory Department, Laboratory for Cheminformatics, National Institute of Chemistry, Hajdrihova 19, SI-1001 Ljubljana (Slovenia), [b] Department of Pharmaceutical Chemistry, Faculty of Pharmacy, University of Ljubljana, Aškerčeva cesta 7, SI-1000 Ljubljana (Slovenia);

Table of contents

Representatives of NBTI antibacterials targeting topoIV enzymes S3

Superposition of co-crystal ligands from DNA gyrase/DNA/NBTI complexes S10

Comparison of GyrA and ParC amino acid sequences of various Gram-positive and Gram-negative bacteria S11

References S12

Representatives of NBTI antibacterials targeting topoIV enzymes

Table S1. Comparison of topoIV inhibition data for the most representative Gram-positive (e.g., *S. aureus*) and Gram-negative (e.g., *E. coli*) bacterial pathogens for a panel of selected NBTIs.

ID	Structure	topoIV IC_{50} [μM] [a,b,c,d]		Ref. (No. of ref. in the paper)
		S. aureus	*E. coli*	
**1**	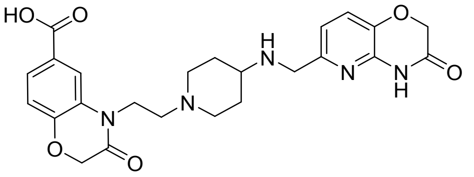	ND	4.680	1 (53)
**2**	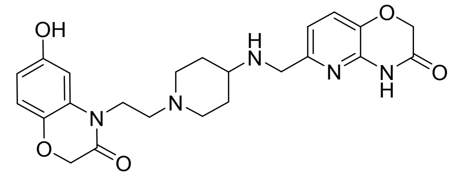	ND	0.036	1 (53)
**3**	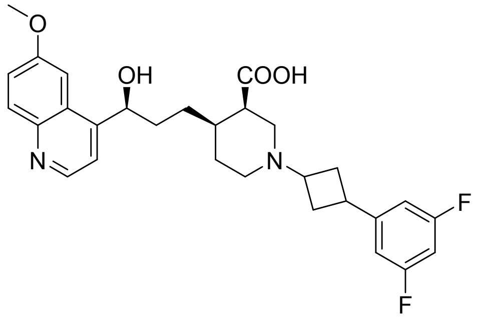	15	ND	2 (42)
**4**	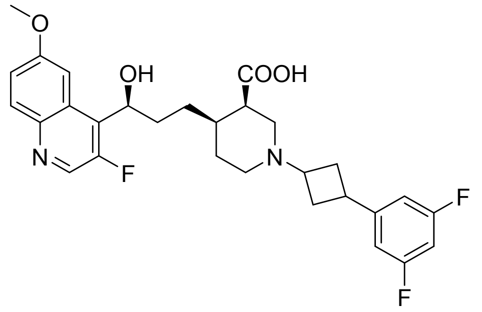	6.25	ND	3 (36)
**5**	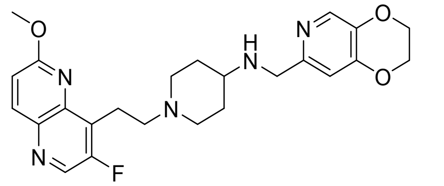	ND	0.0013	4 (50)
**6**	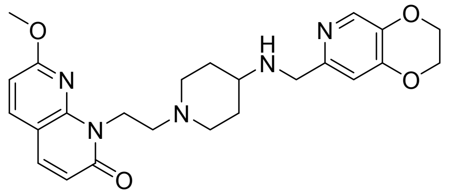	ND	0.002	4 (50)

**7**	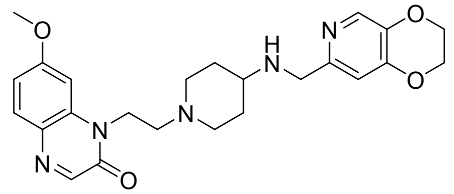	ND	0.005	4<br>(50)
**8**	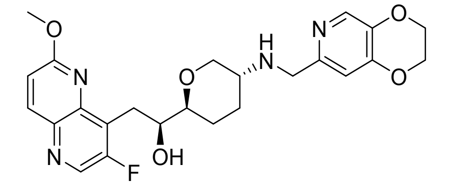	8	0.125	5<br>(48)
**9**	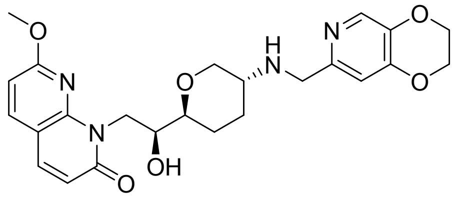	ND	0.125	6<br>(49)
**10**	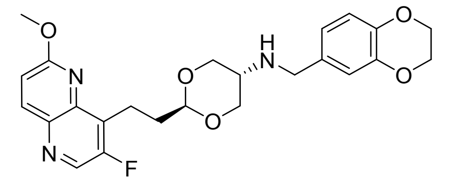	0.98	ND	7<br>(43)
**11**	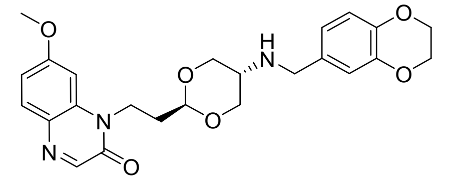	>100	ND	7<br>(43)
**12**	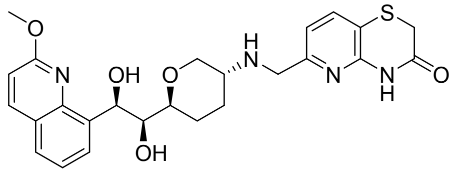	0.5	0.125	8<br>(37)
**13**	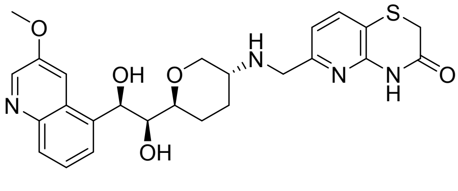	0.5	0.125	8<br>(37)
**14**	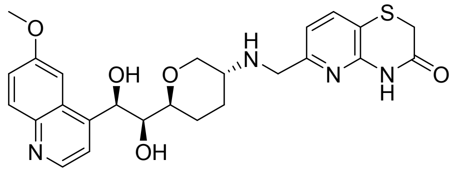	8	0.125	8<br>(37)
**15**	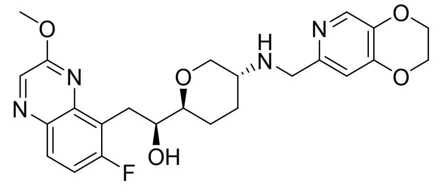	8	0.125	5<br>(48)

**16**	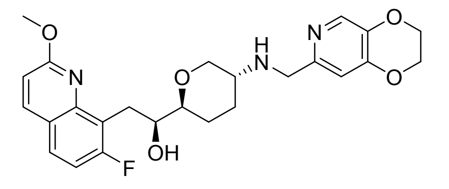	8	0.5	5<br>(48)
**17**	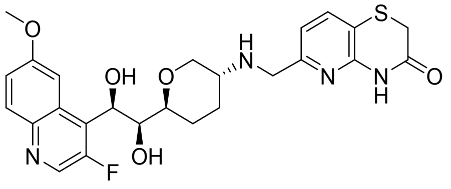	8	0.125	8<br>(37)
**18**	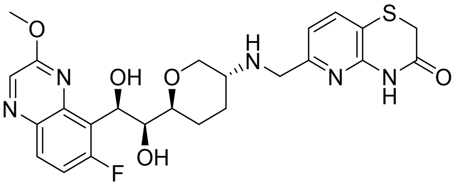	2	0.125	8<br>(37)
**19**	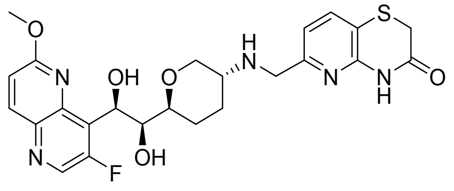	0.5	0.03	8<br>(37)
**20**	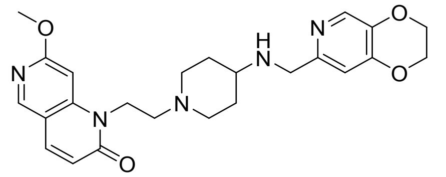	ND	0.028	4<br>(50)
**21**	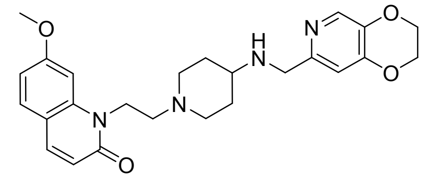	ND	0.022	4<br>(50)
**22**	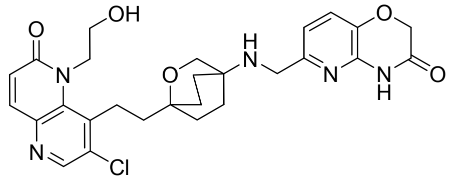	12.8	0.22	9<br>(56)
**23**	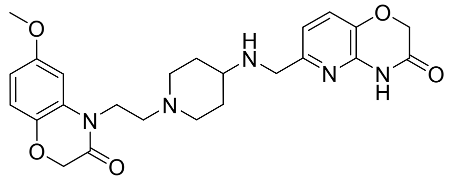	ND	0.005	4<br>(50)
**24**	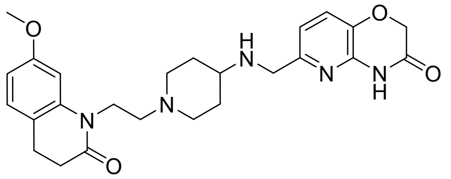	ND	0.78	4<br>(50)

**25**	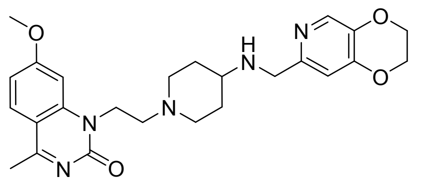	ND	0.082	4<br>(50)
**26**	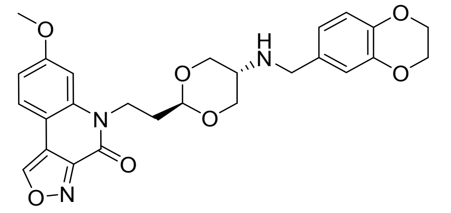	>200	ND	7<br>(43)
**27**	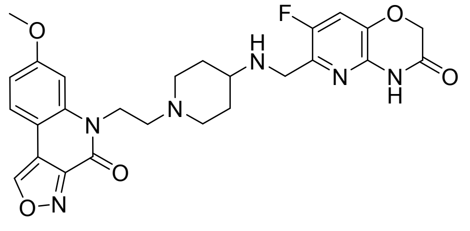	ND	0.10	10<br>(47)
**28**	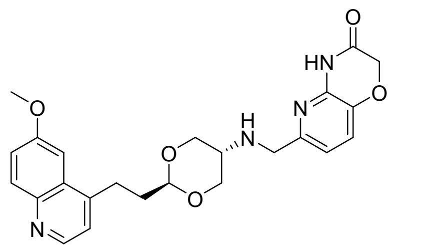	>100	ND	11<br>(45)
**29**	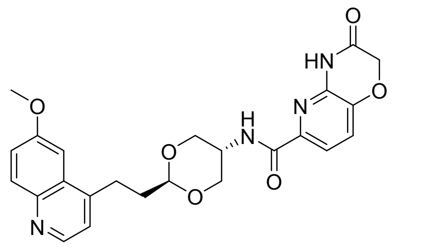	0.19	ND	12<br>(46)
**30**	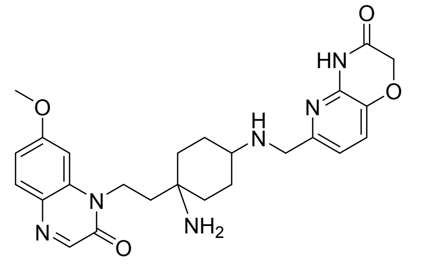	ND	0.002	13<br>(52)
**31**	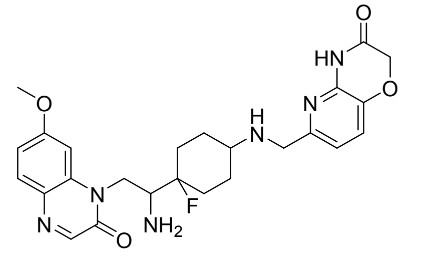	ND	0.005	13<br>(52)

**32**	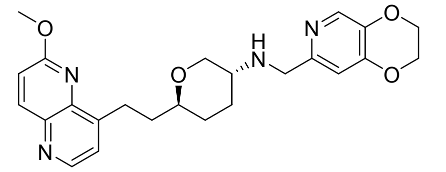	>8	0.125	8<br>(37)
**33**	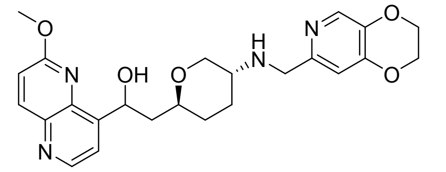	>8	0.125	8<br>(37)
**34**	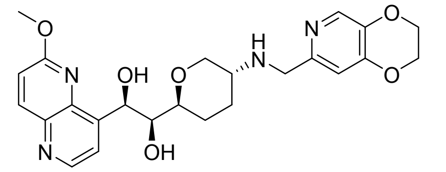	8	0.125	8<br>(37)
**35**	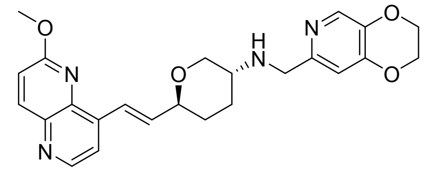	>8	0.125	8<br>(37)
**36**	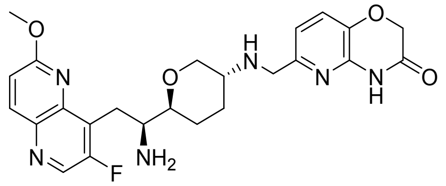	ND	0.03	6<br>(49)
**37**	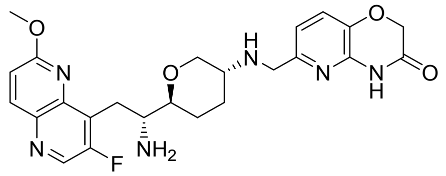	ND	0.125	6<br>(49)
**38**	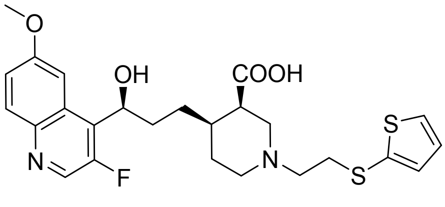	18[b]<br>0.3[c]	2.2[b]<br>0.2[c]	14<br>(19)
**39**	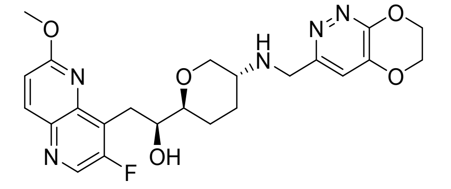	32	2	5<br>(48)
**40**	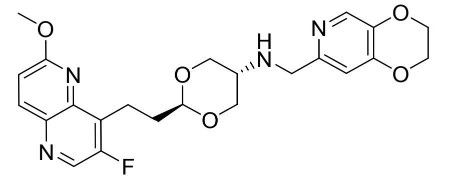	18	ND	7<br>(43)

**41**	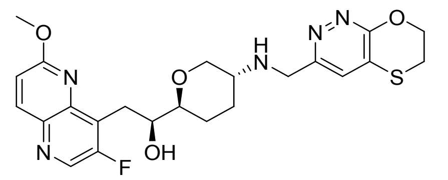	8	0.125	5<br>(48)
**42**	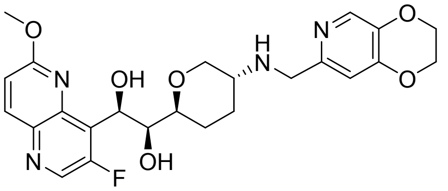	8	0.125	8<br>(37)
**43**	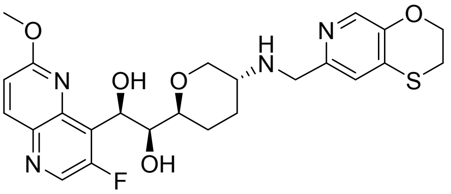	8	0.125	8<br>(37)
**44**	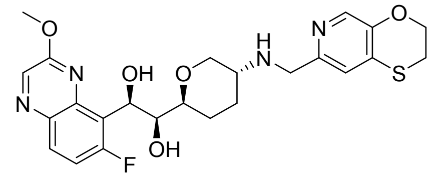	8	0.125	8<br>(37)
**45**	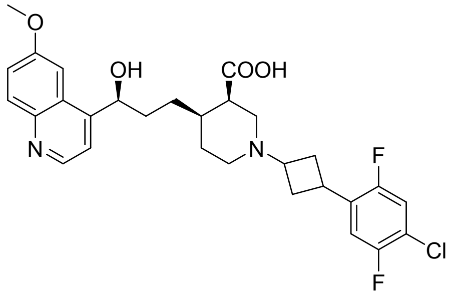	120	ND	2<br>(42)
**46**	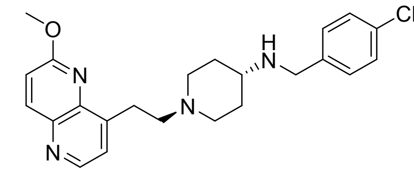	16.37	0.031	15<br>(38)
**47**	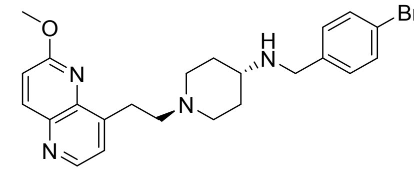	7.22	0.042	15<br>(38)
**48**	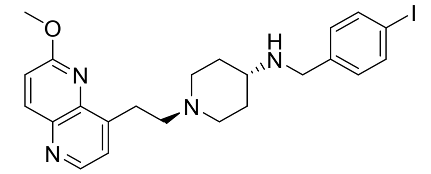	2.13	0.021	15<br>(38)

**49**	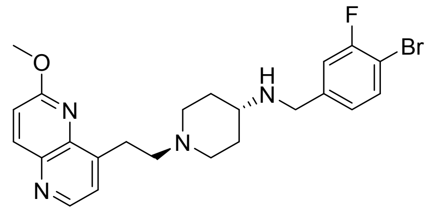	0.24	0.105	16 (41)
**50**	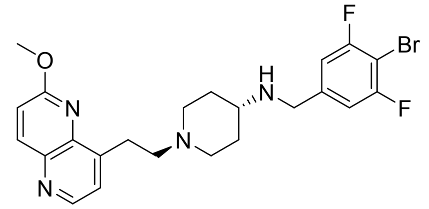	0.05	0.033	16 (41)

[a]ATPase activity assay: references 1,4,13; [b]topoIV Relaxation Assay: references 5,6,8,14; [c]topoIV Relaxation High Throughput Plate Assay: references 15,16; [d]topoIV Decatenation Assay: references 2,3,7,9,11,12,14; ND = not determined.

Superposition of co-crystal ligands from DNA gyrase/DNA/NBTI complexes

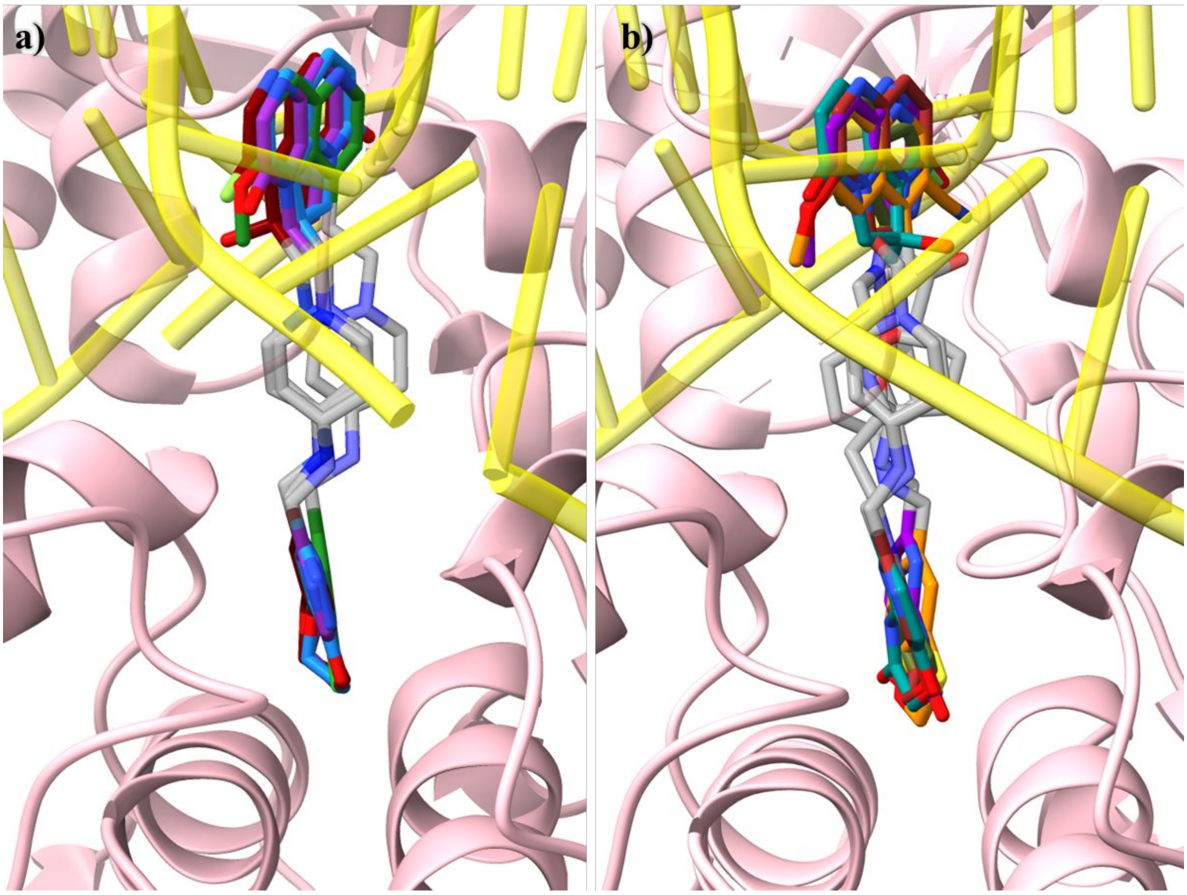

Figure S1. Structural superposition of the co-crystal ligands from available *S. aureus* DNA gyrase-DNA-NBTI complexes. a) Central base pairs are thymine (T) and adenine (A). The ligand from complex with PDB ID 6Z1A[17] is in forest green, 4BUL[18] in dark red, 5IWI[19] in dodger blue, and 6QTK[20] in light violet. b) The central DNA base pairs are guanine (G) and cytosine (C). The ligand from complex with PDB ID 2XCS[21] is in dark orange, 4PLB[22] in dark violet, 6FM4[23] in dark olive green, 6QTP[20] in brown, and 5BS3 in dark cyan. LHS and RHS fragments are colored with the mentioned colors, while linker moieties are in transparent gray. DNA gyrase and DNA are in ribbon presentation and colored in pink and yellow, respectively.

Comparison of GyrA and ParC amino acid sequences of various Gram-positive and Gram-negative bacteria

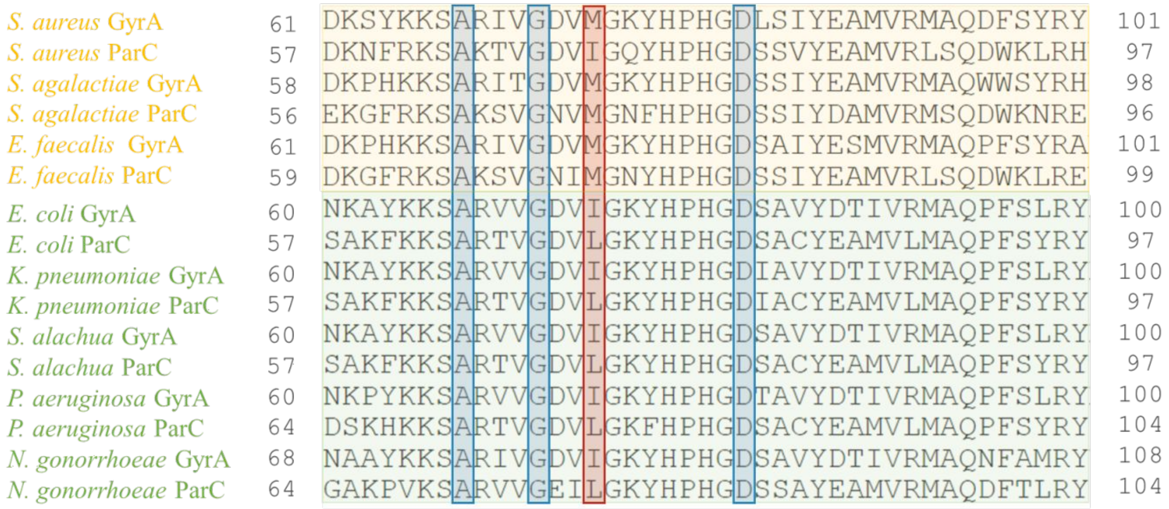

Figure S2. Comparison of amino acid sequences of DNA gyrase GyrA and topoIV ParC subunits from various Gram-positive (*S. aureus*, *S. agalactiae*, *E. faecalis*, in yellow) and Gram-negative bacteria (*E. coli, K. pneumoniae, S. alachua, P. aeruginosa, N. gonorrhoeae,* in green). The key amino acid residues for binding of NBTIs are highlighted in blue and red.

References

(1) Geng, B.; Comita-Prevoir, J.; Eyermann, C. J.; Reck, F.; Fisher, S. Exploring Left-Hand-Side Substitutions in the Benzoxazinone Series of 4-Amino-Piperidine Bacterial Type IIa Topoisomerase Inhibitors. *Bioorganic Med. Chem. Lett.* **2011**, *21* (18), 5432–5435.

(2) Mitton-Fry, M. J.; Brickner, S. J.; Hamel, J. C.; Brennan, L.; Casavant, J. M.; Chen, M.; Chen, T.; Ding, X.; Driscoll, J.; Hardink, J.; Hoang, T.; Hua, E.; Huband, M. D.; Maloney, M.; Marfat, A.; McCurdy, S. P.; McLeod, D.; Plotkin, M.; Reilly, U.; Robinson, S.; Schafer, J.; Shepard, R. M.; Smith, J. F.; Stone, G. G.; Subramanyam, C.; Yoon, K.; Yuan, W.; Zaniewski, R. P.; Zook, C. Novel Quinoline Derivatives as Inhibitors of Bacterial DNA Gyrase and Topoisomerase IV. *Bioorganic Med. Chem. Lett.* **2013**, *23* (10), 2955–2961.

(3) Mitton-Fry, M. J.; Brickner, S. J.; Hamel, J. C.; Barham, R.; Brennan, L.; Casavant, J. M.; Ding, X.; Finegan, S.; Hardink, J.; Hoang, T.; Huband, M. D.; Maloney, M.; Marfat, A.; McCurdy, S. P.; McLeod, D.; Subramanyam, C.; Plotkin, M.; Reilly, U.; Schafer, J.; Stone, G. G.; Uccello, D. P.; Wisialowski, T.; Yoon, K.; Zaniewski, R.; Zook, C. Novel 3-Fluoro-6-Methoxyquinoline Derivatives as Inhibitors of Bacterial DNA Gyrase and Topoisomerase IV. *Bioorganic Med. Chem. Lett.* **2017**, *27* (15), 3353–3358.

(4) Reck, F.; Alm, R.; Brassil, P.; Newman, J.; DeJonge, B.; Eyermann, C. J.; Breault, G.; Breen, J.; Comita-prevoir, J.; Cronin, M.; Davis, H.; Ehmann, D. E.; Galullo, V.; Geng, B.; Grebe, T.; Morningstar, M.; Walker, P.; Hayter, B.; Fisher, S. Novel N-Linked Aminopiperidine Inhibitors of Bacterial Topoisomerase Type II: Broad-Spectrum Antibacterial Agents with Reduced HERG Activity. *J. Med. Chem.* **2011**, *54* (22), 7834–7847.

(5) Surivet, J. P.; Zumbrunn, C.; Rueedi, G.; Bur, D.; Bruyère, T.; Locher, H.; Ritz, D.; Seiler, P.; Kohl, C.; Ertel, E. A.; Hess, P.; Gauvin, J. C.; Mirre, A.; Kaegi, V.; Dos Santos, M.; Kraemer, S.; Gaertner, M.; Delers, J.; Enderlin-Paput, M.; Weiss, M.; Sube, R.; Hadana, H.; Keck, W.; Hubschwerlen, C. Novel Tetrahydropyran-Based Bacterial Topoisomerase Inhibitors with Potent Anti-Gram Positive Activity and Improved Safety Profile. *J. Med. Chem.* **2014**, *58* (2), 927–942.

(6) Surivet, J. P.; Zumbrunn, C.; Bruyère, T.; Bur, D.; Kohl, C.; Locher, H. H.; Seiler, P.; Ertel, E. A.; Hess, P.; Enderlin-Paput, M.; Enderlin-Paput, S.; Gauvin, J. C.; Mirre, A.; Hubschwerlen, C.; Ritz, D.; Rueedi, G. Synthesis and Characterization of Tetrahydropyran-Based Bacterial Topoisomerase Inhibitors with Antibacterial Activity against Gram-Negative Bacteria. *J. Med. Chem.* **2017**, *60* (9), 3776–3794.

(7) Li, L.; Okumu, A. A.; Nolan, S.; English, A.; Vibhute, S.; Lu, Y.; Hervert-Thomas, K.; Seffernick, J. T.; Azap, L.; Cole, S. L.; Shinabarger, D.; Koeth, L. M.; Lindert, S.; Yalowich, J. C.; Wozniak, D. J.; Mitton-Fry, M. J. 1,3-Dioxane-Linked Bacterial Topoisomerase Inhibitors with Enhanced Antibacterial Activity and Reduced HERG Inhibition. *ACS Infect. Dis.* **2019**, *5* (7), 1115–1128.

(8) Surivet, J. P.; Zumbrunn, C.; Rueedi, G.; Hubschwerlen, C.; Bur, D.; Bruyère, T.; Locher, H.; Ritz, D.; Keck, W.; Seiler, P.; Kohl, C.; Gauvin, J. C.; Mirre, A.; Kaegi, V.; Dos Santos, M.; Gaertner, M.; Delers, J.; Enderlin-Paput, M.; Boehme, M. Design, Synthesis, and Characterization of Novel Tetrahydropyran-Based Bacterial Topoisomerase Inhibitors with Potent Anti-Gram-Positive Activity. *J. Med. Chem.* **2013**, *56* (18), 7396–7415.

(9) Tan, C. M.; Gill, C. J.; Wu, J.; Toussaint, N.; Yin, J.; Tsuchiya, T.; Garlisi, C. G.; Kaelin, D.; Meinke, P. T.; Miesel, L.; Olsen, D. B.; Lagrutta, A.; Fukuda, H.; Kishii, R.; Takei, M.; Oohata, K.; Takeuchi, T.; Shibue, T.; Takano, H.; Nishimura, A.; Fukuda, Y.; Singh, S. B. In Vitro and in Vivo Characterization of the Novel Oxabicyclooctane-Linked Bacterial Topoisomerase Inhibitor AM-8722, a Selective, Potent Inhibitor of Bacterial DNA Gyrase. *Antimicrob. Agents Chemother.* **2016**, *60* (8), 4830–4839.

(10) Charrier, C.; Salisbury, A.-M.; Savage, V. J.; Duffy, T.; Moyo, E.; Chaffer-Malam, N.; Ooi, N.; Newman, R.; Cheung, J.; Metzger, R.; McGarry, D.; Pichowicz, M.; Sigerson, R.; Cooper, I. R.; Nelson, G.; Butler, H. S.; Craighead, M.; Ratcliffe, A. J.; Best, S. A.; Stokes, N. R. Novel Bacterial Topoisomerase Inhibitors with Potent Broad-Spectrum Activity against Drug-Resistant Bacteria Skin and Skin Structure Infections. *Antimicrob. Agents Chemother.* **2017**, *61* (5), 1–34.

(11) Li, L.; Okumu, A.; Dellos-Nolan, S.; Li, Z.; Karmahapatra, S.; English, A.; Yalowich, J. C.; Wozniak, D. J.; Mitton-Fry, M. J. Synthesis and Anti-Staphylococcal Activity of Novel Bacterial Topoisomerase Inhibitors with a 5-Amino-1,3-Dioxane Linker Moiety. *Bioorganic Med. Chem. Lett.* **2018**, *28* (14), 2477–2480.

(12) Lu, Y.; Papa, J. L.; Nolan, S.; English, A.; Seffernick, J. T.; Shkolnikov, N.; Powell, J.; Lindert, S.; Wozniak, D. J.; Yalowich, J.; Mitton-Fry, M. J. Dioxane-Linked Amide Derivatives as Novel Bacterial Topoisomerase Inhibitors against Gram-Positive Staphylococcus Aureus. *ACS Med. Chem. Lett.* **2020**, *11* (12), 2446–2454.

(13) Reck, F.; Ehmann, D. E.; Dougherty, T. J.; Newman, J. V.; Hopkins, S.; Stone, G.; Agrawal, N.; Ciaccio, P.; McNulty, J.; Barthlow, H.; O'Donnell, J.; Goteti, K.; Breen, J.; Comita-Prevoir, J.; Cornebise, M.; Cronin, M.; Eyermann, C. J.; Geng, B.; Carr, G. R.; Pandarinathan, L.; Tang, X.; Cottone, A.; Zhao, L.; Bezdenejnih-Snyder, N. Optimization of Physicochemical Properties and Safety Profile of Novel Bacterial Topoisomerase Type II Inhibitors (NBTIs) with Activity against Pseudomonas Aeruginosa. *Bioorganic Med. Chem.* **2014**, *22* (19), 5392–5409.

(14) Black, M. T.; Stachyra, T.; Platel, D.; Girard, A. M.; Claudon, M.; Bruneau, J. M.; Miossec, C. Mechanism of Action of the Antibiotic NXL101, a Novel Nonfluoroquinolone Inhibitor of Bacterial Type II Topoisomerases. *Antimicrob. Agents Chemother.* **2008**, *52* (9), 3339–3349.

(15) Kolarič, A.; Kokot, M.; Hrast, M.; Weiss, M.; Zdovc, I.; Trontelj, J.; Žakelj, S.; Anderluh, M.; Minovski, N. A Fine-Tuned Lipophilicity / Hydrophilicity Ratio Governs Antibacterial Potency and Selectivity of Bifurcated Halogen. *Antibiotics* **2021**, *10* (7), 862.

(16) Kokot, M.; Weiss, M.; Zdovc, I.; Hrast, M.; Anderluh, M.; Minovski, N. Structurally Optimized Potent Dual-Targeting NBTI Antibacterials with an Enhanced Bifurcated Halogen-Bonding Propensity. *ACS Med. Chem. Lett.* **2021**, *12* (9), 1478–1485.

(17) Kolarič, A.; Germe, T.; Hrast, M.; Stevenson, C. E. M.; Lawson, D. M.; Burton, N. P.; Vörös, J.; Maxwell, A.; Minovski, N.; Anderluh, M. Potent DNA Gyrase Inhibitors Bind Asymmetrically to Their Target Using Symmetrical Bifurcated Halogen Bonds. *Nat. Commun.* **2021**, *12* (1), 1–13.

(18) Miles, T. J.; Hennessy, A. J.; Bax, B.; Brooks, G.; Brown, B. S.; Brown, P.; Cailleau, N.; Chen, D.; Dabbs, S.; Davies, D. T.; Esken, J. M.; Giordano, I.; Hoover, J. L.; Huang, J.; Jones, G. E.; Kusalakumari Sukmar, S. K.; Spitzfaden, C.; Markwell, R. E.; Minthorn, E. A.; Rittenhouse, S.; Gwynn, M. N.; Pearson, N. D. Novel Hydroxyl Tricyclics (e.g., GSK966587) as Potent Inhibitors of Bacterial Type IIA Topoisomerases. *Bioorganic Med. Chem. Lett.* **2013**, *23* (19), 5437–5441.

(19) Miles, T. J.; Hennessy, A. J.; Bax, B.; Brooks, G.; Brown, B. S.; Brown, P.; Cailleau, N.; Chen, D.; Dabbs, S.; Davies, D. T.; Esken, J. M.; Giordano, I.; Hoover, J. L.; Jones, G. E.; Kusalakumari Sukmar, S. K.; Markwell, R. E.; Minthorn, E. A.; Rittenhouse, S.; Gwynn, M. N.; Pearson, N. D. Novel Tricyclics (e.g., GSK945237) as Potent Inhibitors of Bacterial Type IIA Topoisomerases. *Bioorganic Med. Chem. Lett.* **2016**, *26* (10), 2464–2469.

(20) Gibson, E. G.; Bax, B.; Chan, P. F.; Osheroff, N. Mechanistic and Structural Basis for the Actions of the Antibacterial Gepotidacin against Staphylococcus Aureus Gyrase. *ACS Infect. Dis.* **2019**, *5* (4), 570–581.

(21) Bax, B. D.; Chan, P. F.; Eggleston, D. S.; Fosberry, A.; Gentry, D. R.; Gorrec, F.; Giordano, I.; Hann, M. M.; Hennessy, A.; Hibbs, M.; Huang, J.; Jones, E.; Jones, J.; Brown, K. K.; Lewis, C. J.; May, E. W.; Saunders, M. R.; Singh, O.; Spitzfaden, C. E.; Shen, C.; Shillings, A.; Theobald, A. J.; Wohlkonig, A.; Pearson, N. D.; Gwynn, M. N. Type IIA Topoisomerase Inhibition by a New Class of Antibacterial Agents. *Nature* **2010**, *466* (7309), 935–940.

(22) Singh, S. B.; Kaelin, D. E.; Wu, J.; Miesel, L.; Tan, C. M.; Meinke, P. T.; Olsen, D.; Lagrutta, A.; Bradley, P.; Lu, J.; Patel, S.; Rickert, K. W.; Smith, R. F.; Soisson, S.; Wei, C.; Fukuda, H.; Kishii, R.; Takei, M.; Fukuda, Y. Oxabicyclooctane-Linked Novel Bacterial Topoisomerase Inhibitors as Broad Spectrum Antibacterial Agents. *ACS Med. Chem. Lett.* **2014**, *5* (5), 609–614.

(23) Magarò, G.; Prati, F.; Garofalo, B.; Corso, G.; Furlotti, G.; Apicella, C.; Mangano, G.; D'Atanasio, N.; Robinson, D.; Di Giorgio, F. P.; Ombrato, R. Virtual Screening Approach and Investigation of Structure-Activity Relationships to Discover Novel Bacterial Topoisomerase Inhibitors Targeting Gram-Positive and Gram-Negative Pathogens. *J. Med. Chem.* **2019**, *62* (16), 7445–7472.
